# Epigenetic aging mediates the association between life course socioeconomic status and decrements in kidney function across a decade

**DOI:** 10.1007/s11357-025-01728-0

**Published:** 2025-06-12

**Authors:** Agus Surachman, Meera N. Harhay, Rose Ann DiMaria-Ghalili, Anthony S. Zannas, David M. Almeida, Christopher L. Coe

**Affiliations:** 1 Department of Epidemiology and Biostatistics, Dornsife School of Public Health, Drexel University, 3215 Market St, Rm 552, Philadelphia, PA 19104 USA; 2 College of Nursing and Health Professions, Drexel University, Philadelphia, PA USA; 3 Department of Medicine, College of Medicine, Drexel University, Philadelphia, PA USA; 4 Department of Psychiatry, School of Medicine, University of North Carolina at Chapel Hill, Chapel Hill, NC USA; 5 Department of Human Development and Family Studies, College of Health and Human Development, The Pennsylvania State University, University Park, PA USA; 6 Center for Healthy Aging, The Pennsylvania State University, University Park, PA USA; 7 Department of Psychology, University of Wisconsin-Madison, Madison, WI USA

**Keywords:** Epigenetic aging, Geroscience, EGFR, Renal aging, Socioeconomic status

## Abstract

Epigenetic aging measures are novel molecular indicators of biological aging that predict age-related chronic disease. We examined whether several established indices of epigenetic aging mediated the association between life course socioeconomic status (SES) and decrements in kidney function across a decade. Biomarker data were from 252 non-Hispanic (NH) Black and white participants who had consented to genetic analyses in Wave 2 (2004–2009) and 3 (2014–2021) of the Midlife in the United States study (MIDUS). Life course SES included parental education, a proxy of early life SES, and a composite score of adult SES based on the highest education level, household income to poverty line ratio, health insurance coverage, perception of the availability of money to meet needs, and difficulty level paying monthly bills. We included five measures of epigenetic age accelerations (EAA), based on the residuals after each epigenetic clock was regressed on chronological age (Horvath, Horvath blood and skin, Hannum, PhenoAge, and GrimAge) and one measure of the pace of aging (DunedinPACE) obtained during MIDUS 2. Kidney function was based on serum creatinine–based estimated glomerular filtration rate (eGFR), calculated using the CKD-EPI formula (without race adjustment). We calculated absolute decrements in eGFR across 11 years between MIDUS waves 2 and 3. Analyses were adjusted for age, sex, and health-related covariates (currently smoking, obese, hypertension, and insulin resistance). Lower adult SES and accelerated epigenetic aging, especially accelerated GrimAge and faster DunedinPACE pace of aging, mediated the association between lower parental education and larger decrements in eGFR. Accelerated epigenetic aging is associated with larger decrements in kidney function across a decade and may be one of the critical explanatory pathways for the higher burden of chronic kidney disease (CKD) among lower SES individuals.

**Supplementary Information:**

The online version contains supplementary material available at 10.1007/s11357-025-01728-0.

## Introduction

Socioeconomic inequality and systemic racism are known to be fundamental causes of health disparities in the USA [1, 2], including chronic kidney disease (CKD), an umbrella term for various disorders that are associated with a progressive decrement in kidney function. CKD is a leading cause of death in the USA, affecting one in seven US adults (CDC, 2023). Studies have documented that individuals with lower education and income, two important indicators of individual or household-level socioeconomic status (SES), are more likely to experience a higher burden of CKD, faster progression to a worse disease stage, and premature mortality [3–6]. Further, the burden of CKD and detrimental outcomes is much higher among racially minoritized groups, especially Black Americans [4, 7]. The mechanisms linking SES and racially minoritized status to the higher burden of CKD are complex and include multifactorial and multilevel factors, including biological, psychological, and social factors [8]. A better understanding of these pathways and clarification of the underlying mechanisms are needed to improve interventions and rectify the persistent socioeconomic and racial disparities in CKD.

Aging is a critical risk factor for various age-related chronic diseases, including CKD. Renal aging includes intrinsic and anatomical changes within the kidneys and age-related decrements in filtration and clearance [9]. Faster decrements in glomerular filtration rate, a clinical indication of accelerated renal aging, are known to be a sensitive diagnostic marker of progression to CKD [10]. In addition to normative aging, faster decrements in kidney function may indicate renal damage and reflect the complications associated with cardiometabolic risk factors [9]. One of the more significant recent advancements in Geroscience research has been the refinement of new molecular and cellular techniques for investigating epigenetic modifications in DNA regulation and expression, which can be used as temporal milestones of aging and employed as aging clocks to track the progression to senescence and age-related pathophysiology [11]. Epigenetic modifications provide molecular signatures of biological aging [12], and several DNA methylation-based epigenetic aging measures have been developed to characterize accelerated biological aging by comparing chronological and biological age [13]. Recently, newer DNA methylation-based epigenetic aging measures have been developed to predict aging phenotypes, specifically morbidity and mortality [14]. Recent studies have also demonstrated that epigenetic changes in DNA methylation can be employed to identify an elevated risk for CKD [15, 16].

Previous studies have documented that lower SES and racially minoritized status are associated with faster decrements in kidney function among otherwise healthy adults—findings that may help to explain the persistent socioeconomic and racial disparities in CKD prevalence [17–19]. Lower SES is consistently associated with accelerated epigenetic aging [20–22]. There is also evidence that Black relative to white American adults shows faster accelerated epigenetic aging [23], potentially due to the cumulative impact of stress from socioeconomic and racial injustices affecting cellular and molecular pathways associated with health [22]. Little is known, however, if the impact of lower SES and racially minoritized status on faster decrements in kidney function can be explained by accelerated epigenetic aging. The current study investigated whether the association between lower SES and kidney function trajectories was mediated by accelerated epigenetic aging. Further, we explored whether this potential mediational pathway differed in Black and white American adults.

The life course framework is crucial to inform the role of SES and epigenetic aging in kidney function trajectories across adulthood. According to the life course health development perspective of CKD, many developmental events and processes across the lifespan can increase the risk of accelerated decrements in kidney function and progression to CKD [19, 24]. For example, early life SES is an especially important socioeconomic context because kidney development and maturation during fetal development and in childhood can have life-long consequences. Adverse birth outcomes (i.e., preterm birth, low birth weight), which are known to be linked to socioeconomic disadvantage, are associated with a lower nephron endowment at birth and changes in renal programming that increase the later risk for CKD in adulthood, including via other types of physiological dysregulation, such as an increased propensity for atherosclerotic cardiovascular disease [25]. Early life socioeconomic adversity is also generally characterized by exposure to higher levels of psychosocial stress that can lead to a biological embedding of inflammatory physiology and elevated risk for cardiometabolic dysregulation and CKD in adulthood [26–28].

Further, socioeconomic adversity early in life often results in constraints on socioeconomic mobility, leading to the perpetuation of lower SES into adulthood (i.e., lower education and income). The accumulation of socioeconomic disadvantages across the life course is a robust predictor of the risk for CKD [19, 29]. Finally, the new epigenetic indices for analyzing DNA methylation provide robust tools to capture the accumulation of physiological wear-and-tear across the life course, including stressors associated with socioeconomic disadvantages [20, 29, 30]. Prior analyses showed accelerated epigenetic aging may also capture socioeconomic disadvantages across generations [31]. Therefore, epigenetic aging may provide an accurate indication of accelerated aging at the molecular level attributed to life course socioeconomic factors, which, in turn, may differentiate normal from faster kidney aging even among relatively healthy adults.

### The current study

In summary, there is growing evidence that DNA methylation-based indicators of epigenetic aging may have predictive value and validity for understanding the age-related progression to CKD. However, it is not known if they are linked to the socioeconomic and racial disparities in risk for CKD. The current analysis examined the association between life course socioeconomic status, epigenetic aging, and decrements in kidney function across a decade among Black and white American adults. We hypothesized that lower life course SES would be associated with accelerated epigenetic aging and, in turn, faster decrements in kidney function. Given prior evidence that SES and racial minoritized status can accentuate epigenetic aging disparities, we also explored if the mediating role of epigenetic aging on the association between life course SES and decrements in kidney function differed between Black and white American adults.

## Methods

### Data and participants

Data for the current analysis were generated by the Midlife in the United States (MIDUS) study, a longitudinal examination of biopsychosocial factors associated with health and well-being among middle and older adults in the contiguous USA. Extensive details about MIDUS have been presented elsewhere [32]. Here, we provided brief information about the protocols relevant to the current analysis. The MIDUS study was started in 1995 and recruited 7108 participants ages 20–75 through random digit dialing (RDD). Ten years later, the first longitudinal follow-up was conducted (MIDUS 2; 2004–2006), and 4963 participants (ages 28–84) completed a survey assessment (70% longitudinal retention rate). To increase the racial diversity of the participants, an oversample of mainly Black adults was recruited from Milwaukee, WI, and completed the MIDUS 2 Milwaukee (*N* = 592, ages 34–85, 93.4% Black). A protocol for Biomarker assessment was also introduced in the MIDUS 2 (MIDUS 2 Biomarker; 2004–2009). The Biomarker study included participants from the national sample and Milwaukee oversample (*N* = 1255). A third follow-up (MIDUS 3) was completed in 2013–2015. It included 3294 participants (ages 39–93) from the national sample and 389 participants (ages 44–94) from the Milwaukee oversample (*N* = 3683). The MIDUS 3 also provided the first longitudinal follow-up of the MIDUS Biomarker study, with 747 participants completing the protocol (MIDUS 3 Biomarker; 2017–2022). Serum creatinine measures were available to determine estimated glomerular filtration rate (eGFR) values for both MIDUS 2 and 3, while the epigenetic aging information was generated from the initial MIDUS 2 Biomarker assessment. The MIDUS protocol was approved by the Health Sciences Institutional Review Board at the University of Wisconsin (Madison, WI), as well as by the IRBs at Georgetown University (Washington D.C.) and UCLA (Los Angeles, CA). Informed consent was obtained twice, prior to the survey and before the biomarker data collection.

#### Biomarker data collection

The protocols for Biomarker assessment in MIDUS 2 and 3 were identical. Participants were assessed during an overnight visit at one of three clinical research centers based on proximity to their home, either in Madison, WI, Washington, DC, or Los Angeles, CA. Fasted blood samples were collected on the morning of the second day before participants had breakfast to determine serum creatinine levels and to obtain whole blood for the DNA methylation testing. All samples were collected and processed using standardized procedures to ensure consistency. Serum creatinine was determined with an enzymatic colorimetric assay at a CLIA-certified clinical laboratory (UnityPoint Health, Madison, WI). Assays of DNA methylation were conducted at the Social Genomics Core Laboratory (UCLA, Los Angeles, CA).

#### Analytic sample

Data were included from participants who completed the MIDUS 2 and 3 Biomarker protocols with available serum creatinine, had consented to the genetics analyses with available epigenetic aging data, self-identified as non-Hispanic (NH) Black and NH white, and had preserved kidney function (i.e., eGFR ≥ 60 mL/min/1.73 m^2^) during the baseline biomarker data collection in MIDUS 2. Of the 1244 participants who completed the MIDUS 2 Biomarker protocol, 11 did not have serum creatinine values. Of the remaining 1233 participants, 677 completed the follow-up biomarker protocol during MIDUS 3 and had serum creatinine data available. DNA methylation data were not available for 391 of these participants, and thus, they were excluded. Of the 286 eligible participants, 276 self-identified as NH Black or NH white. After excluding participants with eGFR < 60 mL/min/1.73 m^2^ at MIDUS 2 (*n* = 13), the sample was 263. Finally, early life SES information, a primary predictor, was missing for 11 participants. The final analytic sample was 252 adults (NH Black = 62).

### Measures

#### Early life SES

Parental educational attainment was used as a proxy for early life SES (0 = graduated from high school/GED or lower, 1 = higher). It was based on participants’ reports on the highest educational attainment of the fathers (or male head of household). The mother’s (or female head of household) highest school level was used if there was incomplete information on the father’s education.

#### Adult SES

We calculated the total score of socioeconomic status (SES) based on five indicators (range = 0–9), where higher scores represent better SES [19]: (1) highest education level (0 = high school diploma/GED, 1 = some college, 2 = bachelor’s degree or higher); (2) ratio between total household income relative to the poverty line, adjusted for household size (0 = < 300%, 1 = 300– < 600%, 2 = 600% or higher); (3) coverage of health insurance (0 = No, 1 = Yes); (4) perception of availability of money to meet needs (0 = not enough, 1 = just enough, 2 = more money than need); (5) perception of difficulty level paying monthly bills (0 = very/somewhat difficult, 1 = not very difficult, 2 = not at all difficult). Collectively, each of these contributing factors to adult SES composite score had acceptable reliability (Cronbach’s alpha = 0.68). Mean imputation was used to handle missing data for eight participants.

#### DNA methylation-based epigenetic aging

Whole blood samples were collected into a vacutainer with EDTA (Becton Dickinson, Franklin Lakes, US), frozen, and used to acquire DNA during the MIDUS 2 Biomarker phase. After the DNA level was examined to ensure suitable yield and integrity, genome-wide methylation was determined using Illumina Methylation EPIC microarrays. DNA methylation was based on beta values, which were noob-normalized to control for technical sources of variance, registered onto the list of CpG sites assayed on the Illumina Methylation 450 K microarray, and screened for quality control using standard metrics. The following DNA methylation-based markers of epigenetic aging were calculated: Horvath [13], Hannum [33], Skin and Blood [34], PhenoAge [35], GrimAge (Version 2) [36], and DunedinPACE epigenetic pace of aging [37]. For Horvath, Hannum, Skin and Blood, PhenoAge, and GrimAge epigenetic clocks, epigenetic age accelerations (EAAs) were calculated based on residual values after regressing each epigenetic marker on chronological age. Thus, positive values of EAA reflect accelerated biological aging relative to chronological age. The DunedinPACE index captures the pace of aging, in which a value of 1 indicates an equivalent rate of biological aging with respect to chronological time (in a 12-month unit). In contrast, a DunedinPACE value > 1 indicates an accelerated pace of biological aging. While others have suggested that it can be valuable to include genome-wide SNP-based principal components and cell counts when analyzing DNA methylation-based epigenetic aging data [38, 39], this information was not available in the MIDUS epigenetic dataset.

#### Kidney function

Blood was also collected in serum separator tubes, and after clotting and centrifugation, 1 mL serum aliquots were frozen and stored in an ultracold freezer until used to assay creatinine in large batches at a CLIA-certified clinical laboratory (UnityPoint Health, Madison, WI). Serum creatinine (SrCr) was quantified with a Roche Cobas Analyzer. The assay range was 0–30.5 mg/dL, with an inter-assay coefficient of variability (CV) of 0.75% for MIDUS 2. For MIDUS 3, the assay range was 0–122 mg/dL, with an inter-assay CV of 2.14%. SrCr was used to calculate the estimated glomerular filtration rate (eGFR) without race adjustment using the Chronic Kidney Disease Epidemiology Collaboration formula (CKD-EPI; mL/min/1.73 m^2^) [40]. Absolute changes in kidney function across a decade were calculated by subtracting eGFR values in MIDUS 2 from those obtained later in the MIDUS 3 follow-up phase. Thus, a higher change score would indicate a higher decrement in an individual’s renal function across a decade.

#### Covariates

Analyses were adjusted for covariates collected during the MIDUS 2 Biomarker study, including age (years), sex (0 = female, 1 = male), individual variation in the follow-up interval between MIDUS 2 and 3 across participants (years), smoking status (0 = never smoke/currently not smoking; 1 = currently smoking), obesity status (body mass index ≥ 30 kg/m^2^ = 1, otherwise coded as 0), elevated blood pressure (systolic and diastolic BP ≥ 140/90 mm Hg or self-report of physician-diagnosed hypertension = 1, otherwise coded as 0), and evidence of insulin resistance (HbA1c ≥ 6.5% or fasting blood glucose ≥ 126 mg/dl or self-reported diagnosis of type 2 diabetes by a physician = 1, otherwise coded as 0). Fasting blood glucose was determined using an enzymatic colorimetric method (assay range: 2–750 mg/dL; intra-assay and inter-assay CV: 1%) performed at the ARUP Lab (Salt Lake City, UT). Glycosylated hemoglobin (HbA1c) was measured using immunochemiluminescent methods at UnityPoint Health clinical laboratory (Madison, WI; assay range: 2.4–75.8 mg/dL; intra-assay CV: 0.43%; inter-assay CV: 1.1–3.4%).

### Analytic strategy

The analyses were divided into two parts. In the first part, we conducted regression analyses to examine the association between life course SES, each measure of epigenetic aging, and eGFR change across a decade. Second, we examined the mediating role of the indices of epigenetic aging on the association between life course SES and change in eGFR across a decade.

#### Regression analysis on the association between life course SES, epigenetic aging, and decrements in kidney function

First, we examined the association between life course SES and each measure of epigenetic aging in separate regression models. In the least adjusted model (Model 1), parental education was the only predictor of epigenetic aging, adjusted for age and sex. Adult SES was then added to the model to test the association of both early life and contemporaneous SES simultaneously and epigenetic aging (Model 2). In the fully adjusted models (Model 3), health-related factors, including smoking, obesity, elevated blood pressure, and insulin resistance status, were added as additional covariates. As part of our exploratory analyses, we examined the association between life course SES and each measure of epigenetic aging separately between NH Black and NH white adults. We conducted stratified analyses based on race using the same modeling strategies.

Second, we tested the association between life course SES, each measure of epigenetic aging, and decrements in kidney function. The analysis involving each epigenetic aging measure was conducted using separate regression models. In the least adjusted model (Model 1), we examined the association between parental education and decrements in kidney function, adjusted for age, sex, and the time interval between the MIDUS 2 and MIDUS 3 Biomarker protocols. In the subsequent regression model (Model 2), adult SES and an epigenetic aging measure were added to the model. In the fully adjusted model (Model 3), smoking, obesity, elevated blood pressure, and insulin resistance status were added as additional covariates. We also explored the association between life course SES, epigenetic aging, and decrements in kidney function separately for NH Black and NH white adults by conducting stratified analyses based on race using the same modeling strategies.

#### Mediation analyses

**Fig. 1:**
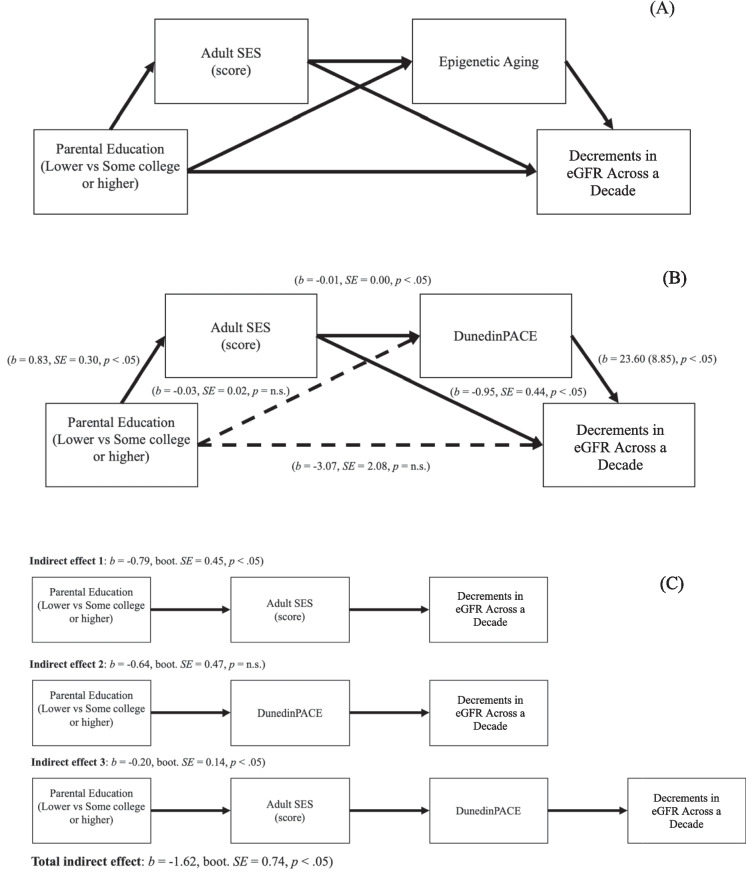
**A** Hypothesized mediation model on the associations among parental education, adult SES, epigenetic aging, and changes in eGFR across a decade. **B** Results from the mediation analysis. Adult SES and DunedinPACE fully mediated the association between lower SES and faster decrements in eGFR across a decade (*N* = 274). **C** Breakdown of the indirect effects from the mediation analysis. There were two significant indirect paths from parental education to changes in eGFR: (1) through adult SES and (2) through adult SES and DunedinPACE. The indirect path from parental education to changes in eGFR through DunedinPACE was not statistically significant. Mediation analysis was adjusted for age, sex, time intervals (years) between MIDUS 2 and 3 Biomarker protocols, smoking, obesity, elevated blood pressure, and insulin resistance status

Mediation analyses were conducted with the PROCESS package in R [41] using 10,000 bootstrapped samples. Mediation analyses were adjusted for all covariates. The hypothesized mediation model is presented in Fig. 1A, and significant associations were based on attaining alpha lower than 0.05. Three indirect effects were tested through which parental education was associated with decrements in kidney function across a decade: (1) through adult SES, (2) through epigenetic aging, and (3) through adult SES and epigenetic aging. Mediation by each epigenetic aging measure was conducted using separate mediation models. Finally, moderated mediation analyses were included to explore whether the mediation by epigenetic aging on the association between life course SES and age-related decrements in eGFR differed between NH Black and NH white groups (see Supplementary Materials for the hypothesized model of moderated mediation, Figure S1). The moderated mediation analyses also tested the same three indirect effects, with indirect effects calculated separately for NH Black and NH white groups. Significant moderated mediation for indirect effects in moderated mediation analyses was based on the index of moderated mediation [42].

## Results

### Descriptive statistics

Descriptive statistics for all variables included in the analyses are presented in Table 1. The mean absolute change in eGFR between MIDUS 2 and MIDUS 3 was 11.43 (*SD* = 15.61) mL/min/1.73 m^2^, indicative of an annual decrement of 1.04 mL/min/1.73 m^2^. NH Black and white participants had similar eGFR values at the first assessment during MIDUS 2. However, the absolute decrements across a decade were larger among NH Black participants (*M* = 15.87, *SD* = 20.66). Slightly over one-tenth of the participants had an eGFR lower than 60 mL/min/1.73 m^2^ during follow-up. The proportion was higher among the NH Black participants (14.5%) than the NH white group (10.5%). NH Black participants also reported higher socioeconomic disadvantages on all SES indicators relative to NH white participants. Finally, NH Black participants evinced faster epigenetic aging than NH white participants on all six indices. Bivariate correlations among variables included in the analyses are provided in Supplementary Materials (Table S1).

**Table 1:** Descriptive statistics of all the variables included in the current analysis (*N* = 252)

	Missing data	All sample (*N* = 252)	NH Black (*n* = 62)	NH white (*n* = 190)
Age in MIDUS 2 (*SD*)	0	53.03 (9.36)	50.87 (8.31)	53.73 (9.59)
Time between MIDUS 2 and 3 (years)	0	11 (1.26)	11.19 (1.17)	10.93 (1.28)
Female (%)	0	57.1	62.9	55.3
Parental education (%)	0			
HS/GED or lower		65.5	79.0	61.1
Some college or higher		34.5	21.0	38.9
Socioeconomic status in MIDUS 2 (score: 0–9)	0	5.33 (2.38)	3.38 (2.06)	5.96 (2.13)
Education (%)				
HS/GED		22.2	38.7	19.5
Some college		28.6	41.9	24.2
Bachelor’s degree or higher		46.8	19.4	55.8
Income to poverty ratio, adjusted for household size (%)				
< 300%		24.2	48.4	16.3
300– < 600%		33.3	33.9	33.2
≥ 600%		40.5	17.7	47.9
Covered by health insurance (%)		90.1	79.0	93.7
Availability of money to meet needs (%)				
Not enough		24.2	54.8	14.2
Just enough		48.4	37.1	52.1
More money		27.0	8.1	33.2
Difficulty level paying bills (%)				
Very/somewhat		33.3	62.9	23.7
Not very		29.0	17.7	32.6
Not at all		37.3	19.4	43.2
Health-related covariates (MIDUS 2)				
Currently smoking	0	19.0	30.6	15.3
Obese	0	38.1	54.8	32.6
Elevated blood pressure	0	43.7	59.7	38.4
Insulin resistance	0	15.9	35.5	9.5
Epigenetic age acceleration (EAA) and pace of aging in MIDUS 2				
EAA Horvath	0	0.00 (3.92)	0.37 (4.26)	− 0.12 (3.80)
EAA Horvath blood and skin	0	0.00 (2.85)	− 0.18 (3.09)	0.06 (2.78)
EAA Hannum	0	0.00 (3.76)	− 1.46 (4.26)	0.48 (3.46)
EAA PhenoAge	0	0.00 (5.71)	0.41 (6.40)	− 0.13 (5.47)
EAA GrimAge	1	0.00 (5.15)	2.68 (5.70)	− 0.88 (4.65)
DunedinPACE pace of aging	0	1.00 (0.13)	1.10 (0.14)	0.97 (0.11)
Kidney Function				
eGFR in MIDUS 2 (mL/min/1.73 m^2^)	0	92.40 (13.92)	95.16 (15.14)	91.51 (13.41)
eGFR in MIDUS 3 (mL/min/1.73 m^2^)	0	80.97 (16.68)	79.28 (20.47)	81.52 (15.27)
Decrements in eGFR (mL/min/1.73 m^2^)	0	11.43 (15.61)	15.87 (20.66)	9.98 (13.31)
eGFR < 60 mL/min/1.73 m^2^ at follow-up (%)	0	11.5	14.5	10.5

*eGFR*, estimated glomerular filtration rate based on serum creatinine, calculated using the CKD-EPI (Chronic Kidney Disease Epidemiology Collaboration) formula without race adjustment. Changes in eGFR were calculated by subtracting eGFR in MIDUS 2 from values in MIDUS 3

### Results from regression analyses on the association between life course SES and epigenetic aging

In the models adjusted for age and sex, higher parental education was associated with slower EAA GrimAge (Table 2A, Model 1; *b* = − 2.05, *SE* = 0.67, 95% *CI* = [− 3.37, − 0.74]) and slower DunedinPACE pace of aging (Table 2A, Model 1; *b* = − 0.07, *SE* = 0.02, 95% *CI* = [− 0.10, − 0.04]), but not the other four epigenetic aging measures (see Supplementary Materials, Table S2). The association between parental educational attainment and DunedinPACE was attenuated but remained significant after adding the adult SES score to the model (Table 2A, Model 2; *b* = − 0.05, *SE* = 0.02, 95% *CI* = [− 0.08, − 0.01]). However, the association between parental education and EAA GrimAge became non-significant after including the adult SES score in the model (Table 2A, Model 2; *b* = − 1.08, *SE* = 0.65, 95% *CI* = [− 2.36, 0.19]). Further, in the fully adjusted model, the association between parental education and DunedinPACE became nonsignificant (Table 2A, Model 3; *b* = − 0.02, *SE* = 0.02, 95% *CI* = [− 0.05, 0.01]).

**Table 2:** Summary from regression analyses on the association between life course SES, epigenetic aging, and decrements in eGFR

**Table 2A. The association between life course SES and epigenetic aging (*N*** **= 252)**								
	Outcome: epigenetic aging							
	Model 1		Model 2		Model 3			
	*B* (*SE*)	95% *CI*	*B* (*SE*)	95% *CI*	*B* (*SE*)		95% *CI*	
**Outcome: EAA GrimAge (*N*** **= 251)**								
Parental education (0 = HS/GED, 1 = higher)	**− 2.05 (0.67)**	**[− 3.37, − 0.74]**	− 1.08 (0.65)	[− 2.36, 0.19]	− 0.37 (0.52)		[− 1.39, 0.66]	
Adult SES (score)			**− 0.78 (0.13)**	**[− 1.03, − 0.52]**	**− 0.40 (0.11)**		**[− 0.62, − 0.18]**	
**Model summary**	*R*^2^ = .07		*R*^2^ = .19		*R*^2^ = .50			
	***F*** **(3, 247) = 6.34**		***F*** **(4, 246) = 14.22**		***F*** **(8, 242) = 30.15**			
**Outcome: DunedinPACE pace of aging (*N*** **= 252)**								
Parental education (0 = HS/GED, 1 = higher)	**− 0.07 (0.02)**	**[− 0.10, − 0.04]**	**− 0.05 (0.02)**	**[− 0.08, − 0.01]**	− 0.02 (0.02)		[− 0.05, 0.01]	
Adult SES (score)			**− 0.02 (0.00)**	**[− 0.03, − 0.01]**	**− 0.01 (0.00)**		**[− 0.02, − 0.00]**	
**Model summary**	*R*^2^ = .07		*R*^2^ = .18		*R*^2^ = .37			
	*F* **(3, 248) = 6.16**		*F* **(4, 247) = 13.48**		***F*** **(8, 243) = 17.52**			
**Table 2B. The association between life course SES, epigenetic aging, and decrements in eGFR across a decade (*N*** **= 252)**								
	Outcome: decrements in eGFR across a decade							
	Model 1		Model 2			Model 3		
	*B* (*SE*)	95% *CI*	*B* (*SE*)	95% *CI*		*B* (*SE*)		95% *CI*
Parental education (0 = HS/GED, 1 = higher)	**− 5.75 (2.03)**	**[− 9.76, − 1.74]**	− 3.62 (2.08)	[− 7.71, 0.48]		− 3.24 (2.07)		[− 7.32, 0.83]
Adult SES (score)			**− 1.13 (0.44)**	**[− 1.99, − 0.26]**		**− 0.97 (0.45)**		**[− 1.84, − 0.09]**
EAA GrimAge			0.25 (0.20)	[− 0.15, 0.64]		0.62 (0.25)		[0.12, 1.12]
**Model summary**	*R*^2^ = .09		*R*^2^ = .13			*R*^2^ = .17		
	***F*** **(4, 247) = 5.99**		***F*** **(6, 244) = 6.12**			***F*** **(10, 240) = 4.98**		
Parental education (0 = HS/GED, 1 = higher)	**− 5.75 (2.03)**	**[− 9.76, − 1.74]**	− 3.14 (2.08)	[− 7.24, 0.96]		− 3.07 (2.08)		[− 7.16, 1.02]
Adult SES (score)			**− 0.93 (0.43)**	**[− 1.79, − 0.08]**		**− 0.95 (0.44)**		**[− 1.82, − 0.08]**
DunedinPACE			**19.27 (7.87)**	**[3.77, 34.77]**		**23.60 (8.85)**		**[6.16, 41.03]**
**Model summary**	*R*^2^ = .09		*R*^2^ = .14			*R*^2^ = .17		
	***F*** **(4, 247) = 5.99**		***F*** **(6, 245) = 6.89**			***F*** **(10, 241) = 5.08**		

Table 2A: Model 1 and 2, adjusted for age and sex; Model 3, adjusted for age, sex, smoking, obese, elevated blood pressure, and insulin resistance status; *B*, unstandardized regression coefficient; *SE*, standard error; *CI*, confidence intervals. Bolded numbers indicate significant association (*p* <.05). Full results from regression analyses are included as part of the Supplementary Materials

Table 2B: Model 1 and 2, adjusted for age, sex, and time intervals (years) between MIDUS 2 and 3 Biomarker protocols; Model 3, adjusted for age, sex, time intervals between MIDUS 2 and 3 Biomarker protocols smoking, obese, elevated blood pressure, and insulin resistance status; *B*, unstandardized regression coefficient; *SE*, standard error; *CI*, confidence intervals. Bolded numbers indicate significant association (*p* <.05). Full results from regression analyses are included as part of the Supplementary Materials

**Fig. 2:**
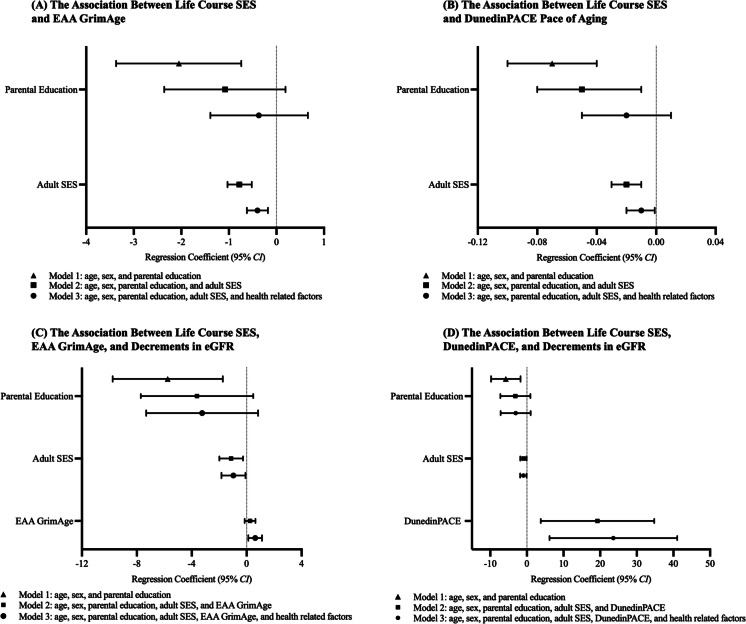
Forest plots based on the unstandardized regression estimates: **A** the association between life course SES and EAA GrimAge; **B** the association between life course SES and DunedinPACE pace of aging; **C** the association between life course SES, EAA GrimAge, and decrements in eGFR; and **D** the association between life course SES, DunedinPACE pace of aging, and decrements in eGFR

Adjusted for age and sex, lower adult SES was associated with faster EAA GrimAge (Table 2, Model 2 A; *b* = − 0.78, *SE* = 0.13, 95% *CI* = [− 1.03, − 0.52]) and DunedinPACE pace of aging (Table 2, Model 2 A; *b* = − 0.02, *SE* = 0.00, 95% *CI* = [− 0.03, − 0.01]). Like parental education, adult SES was not significantly associated with other epigenetic aging measures (see Supplementary Materials, Table S2). In the fully adjusted model (including smoking, obesity, elevated BP, and insulin resistance status in the model), adult SES remained significantly associated with both EAA GrimAge (Table 2A, Model 3; *b* = − 0.40, *SE* = 0.11, 95% *CI* = [− 0.62, − 0.18]) and DunedinPACE pace of aging (Table 2A, Model 3; *b* = − 0.01, *SE* = 0.00, 95% *CI* = [− 0.02, − 0.00]). The summary of findings on the association between life course and epigenetic aging is presented in Fig. 2A (EAA GrimAge) and B (DunedinPACE pace of aging).

### Results from regression analyses on the association between life course SES, epigenetic aging, and decrements in kidney function

Given that EAA GrimAge and DunedinPACE pace of aging were the only two indices of epigenetic aging associated with parental education and adult SES, we focused on those measures of epigenetic aging and their associations with changes in eGFR across a decade. The results from this regression analysis are presented in Table 3 (see Supplementary Materials, Table S3, for full results). Lower parental education was associated with larger decrements in eGFR across a decade (Table 2B, Model 1; *b* = − 5.75, *SE* = 2.03, 95% *CI* = [− 9.76, − 1.74]). However, the association between parental education and decrements in eGFR became non-significant after adding adult SES and epigenetic aging indices to the models (see Table 2B, Model 2). In the fully adjusted model, adult SES (Table 2B, Model 3; *b* = − 0.97, *SE* = 0.45, 95% *CI* = [− 1.84, − 0.09]), EAA GrimAge (Table 2B, Model 3; *b* = 0.62, *SE* = 0.25, 95% *CI* = [0.12, 1.12]), and DunedinPACE (Table 2B; *b* = 23.60, *SE* = 8.85, 95% *CI* = [6.16, 41.03]) were significantly associated with decrements in eGFR. Specifically, lower adult SES scores and faster epigenetic aging were associated with larger decrements in eGFR across a decade. A summary of findings on the association between life course SES, epigenetic aging (i.e., EAA GrimAge and DunedinPACE), and decrements in eGFR is presented in Fig. 2A–D.

**Table 3:** Results from mediation analyses on the association between socioeconomic status, epigenetic aging, and decrements in kidney function across a decade (*N* = 252)

	Adult SES		EAA GrimAge		Decrements in eGFR	
	*B* (*SE*)	95% *CI*	*B* (*SE*)	95% *CI*	*B* (*SE*)	95% *CI*
Parental education	**0.84 (0.30)**	**[0.24, 1.43]**	− 0.50 (0.53)	[− 1.54, 0.54]	− 3.24 (2.07)	[− 7.32, 0.83]
Adult SES			** − 0.40 (0.11)**	**[− 0.61, − 0.18]**	** − 0.97 (0.44)**	**[− 1.84, − 0.09]**
EAA GrimAge					**0.62 (0.25)**	**[0.13, 1.12]**
**Model summary**	*R*^2^ = .21		*R*^2^ = .50		*R*^2^ = .17	
	***F* (8, 242) = 8.20**		***F* (9, 241) = 27.07**		***F* (10, 240) = 4.98**	
**Indirect effects**			**Effect (boot. *SE*)**		**95% *CI* (bootstrapped)**	
Parental education ➔ adult SES ➔ decrements in eGFR			** − 0.81 (0.44)**		**[− 1.77, − 0.06]**	
Parental education ➔ EAA GrimAge ➔ decrements in eGFR			− 0.31 (0.38)		[− 1.23, 0.28]	
Parental education ➔ adult SES ➔ EAA GrimAge ➔ decrements in eGFR			− 0.21 (0.17)		[− 0.62, 0.00]	
Total indirect effects			** − 1.33 (0.67)**		**[− 2.84, − 0.22]**	
	Adult SES		DunedinPACE		Decrements in eGFR	
	*B* (*SE*)	95% *CI*	*B* (*SE*)	*B* (*SE*)	95% *CI*	*B* (*SE*)
Parental education	0.83 (0.30)	[0.23, 1.42]	− 0.03 (0.02)	[− 1.54, 0.54]	− 3.07 (2.08)	[− 7.16, 1.02]
Adult SES			** − 0.01 (0.00)**	**[− 0.02, − 0.04]**	** − 0.95 (0.44)**	**[− 1.82, − 0.08]**
DunedinPACE					**23.60 (8.85)**	**[6.16, 41.03]**
**Model summary**	*R*^2^ = .21		*R*^2^ = .37		*R*^2^ = .17	
	***F* (8, 243) = 8.24**		***F* (9, 242) = 15.69**		***F* (10, 241) = 5.08**	
**Indirect effects**			**Effect (boot. *SE*)**		**95% *CI* (bootstrapped)**	
Parental education ➔ adult SES ➔ decrements in eGFR			** − 0.79 (0.45)**		**[− 1.79, − 0.05]**	
Parental education ➔ DunedinPACE ➔ decrements in eGFR			− 0.64 (0.47)		[− 1.75, 0.04]	
Parental education ➔ adult SES ➔ DunedinPACE ➔ decrements in eGFR			** − 0.20 (0.14)**		**[− 0.55, − 0.02]**	
Total indirect effects			** − 1.62 (0.74)**		**[− 3.26, − 0.39]**	

Analyses adjusted for age, sex, time intervals (years) between MIDUS 2 and 3 Biomarker protocols, smoking, obesity, elevated blood pressure, and insulin resistance status

*B*, unstandardized regression coefficient; *SE*, standard error; *boot. SE*, bootstrapped standard error; *CI*, confidence interval; *EAA*, epigenetic age acceleration. Bolded numbers indicate significant association (*p* <.05)

### Results from mediation analyses

Mediation analyses were conducted to formally test the mediating roles of EAA GrimAge and DunedinPACE on the association between life course SES and age-related decrements in eGFR. Table 3 provides the results from the mediation analysis involving DunedinPACE pace of aging (see Supplementary Materials, Table S4, for full results). The results from the mediation analysis involving EAA GrimAge are provided in Supplementary Materials (Table S5).

#### Mediation by DunedinPACE pace of aging

The mediation model indicated that the pathway from parental education to decrements in eGFR across a decade was mediated by adult SES and DunedinPACE pace of aging. Lower parental education was associated with lower adult SES, which in turn was associated with faster DunedinPACE pace of aging. Both lower adult SES and faster DunedinPACE pace of aging were associated with larger decrements in eGFR across a decade (see Fig. 1B). Two indirect pathways were significant (see Fig. 1C; total indirect effects: effect =  − 1.62, bootstrapped *SE* = 0.74, 95% *CI* (bootstrapped) = [− 3.26, − 0.39])): (1) from lower parental education to lower adult SES to larger decrements in eGFR (effect =  − 0.79, bootstrapped *SE* = 0.45, 95% *CI* (bootstrapped) = [− 1.79, − 0.05]) and (2) from lower parental education to lower adult SES to faster DunedinPACE to larger decrements in eGFR (effect =  − 0.20, bootstrapped *SE* = 0.14, 95% *CI* (bootstrapped) = [− 0.55, − 0.02]). The indirect effect from lower parental education to faster DunedinPACE to larger decrements in eGFR was not statistically significant (effect =  − 0.64, bootstrapped *SE* = 0.47, 95% *CI* (bootstrapped) = [− 1.75, 0.04]).

#### Mediation by EAA GrimAge

Similarly, the pathway from parental education to age-related decrements in eGFR was also mediated by adult SES and EAA GrimAge. Lower parental education was associated with lower adult SES, which, in turn, was associated with accelerated EAA GrimAge. Both lower adult SES and faster EAA GrimAge were associated with larger decrements in eGFR. However, only one of the indirect paths was statistically significant (total indirect effects: effect =  − 1.33, bootstrapped *SE* = 0.67, 95% *CI* (bootstrapped) = [− 2.84, − 0.22]), which is from lower parental education to lower adult SES to larger decrements in eGFR (effect =  − 0.81, bootstrapped *SE* = 0.44, 95% *CI* (bootstrapped) = [− 1.77, − 0.06]). The indirect path from lower parental education to lower adult SES to accelerated EAA GrimAge to larger decrements in eGFR (effect =  − 0.21, bootstrapped *SE* = 0.17, 95% *CI* (bootstrapped) = [− 0.62, 0.00]) or the indirect path from lower parental education to accelerated EAA GrimAge to larger decrements in eGFR was not significant (effect =  − 0.31, bootstrapped *SE* = 0.38, 95% *CI* (bootstrapped) = [− 1.23, 0.28]).

### Exploratory analyses: racial differences in the associations among life course SES, epigenetic aging, and decrements in eGFR across a decade

Results from race-stratified analyses on the association among life course SES, epigenetic aging, and decrements in eGFR across a decade are presented in Supplementary Materials (Tables S7, S8, and S9). Race significantly moderated the mediation model from parental education to decrements in eGFR across a decade through adult SES and EAA GrimAge (see Supplementary Materials, Figure S2). The interaction between race and EAA GrimAge on the age-related decrements in eGFR was statistically significant (see Supplemental Table 10 for full results; *b* = − 1.03, *SE* = 0.42, 95% *CI* = [− 1.85, − 0.22]). The moderation by race was statistically significant on the indirect path from lower parental education to lower adult SES to accelerated EAA GrimAge to larger decrements in eGFR (index of moderated mediation: effect = 0.34, bootstrapped *SE* = 0.25, 95% *CI* = [0.01, 0.95]). This indirect effect was significant only among the NH white participants (effect =  − 0.32, bootstrapped *SE* = 0.20, 95% *CI* [bootstrapped] = [− 0.79, − 0.05]), but not for the NH Black group (effect = 0.03, bootstrapped *SE* = 0.20, 95% *CI* (bootstrapped) = [− 0.40, 0.44]). Finally, the interaction between race and DunedinPACE pace of aging on the age-related decrements in eGFR was not statistically significant (see Supplementary Materials, Table S11 for full results; *b* = − 29.02, *SE* = 17.23, 95% *CI* = [− 62.96, 4.92]). Overall, none of the indirect paths involving the DunedinPACE index of epigenetic age was moderated by race.

## Discussion

Research in the field of Geroscience has contributed significantly to our understanding of the biological underpinnings of aging [11]. The possibility of extending the length of healthspan by slowing down the rate of aging is a promising future goal for addressing societal concerns associated with the growing number of older adults and the burgeoning public health and fiscal burdens of age-related chronic diseases [43]. Achieving these goals requires an understanding of how social, psychological, and behavioral factors contribute to accelerating biological aging and increasing the risk for age-related chronic diseases [43, 44]. Given that adverse social, psychological, and behavioral factors of accelerated aging are systematically structured based on societal stratification criteria, this critical knowledge would be beneficial to foster equity in healthy aging [45].

Our analysis examined whether DNA methylation-based epigenetic aging, robust indicators of epigenetic alterations associated with aging, and predictors for morbidity and mortality [14] mediated the association between life course SES and decrements in eGFR, a robust risk factor for CKD, a significant public health problem. Because life course socioeconomic disadvantages significantly account for racial disparities in CKD, we explored if this mediational association differed between NH Black and white American adults. We found that epigenetic aging measures, specifically accelerated EAA GrimAge and faster DunedinPACE pace of aging, mediated the association between lower life course SES and larger decrements in eGFR across a decade. Further, we found that the mediation by EAA GrimAge was conditional on race. The significant association was evident only among the NH white adults in the MIDUS study.

The more recent epigenetic aging measures have been refined to better predict healthspan by including clinical biomarkers and mortality endpoints in their algorithm development, such as GrimAge [36]. In particular, the sensitivity of GrimAge may reflect that the training program for this epigenetic clock was based on several blood proteins, such as C-reactive proteins (CRP and plasminogen activation inhibitor-1 (PAI-1) that are likely to be associated with renal health [36]. In the case of DunedinPACE, the pace of aging measure was developed as a predictor of the within-individual rate of decline in organ-system integrity [37]. Evidence for age acceleration on both epigenetic clocks has been linked to social, psychological, and behavioral factors associated with age-related chronic diseases and mortality. For example, accelerated GrimAge and faster DunedinPACE mediated the association between lower SES and poorer cognitive performance [46], exposure to environmental toxins and CVD [47], lower Life’s Essential 8 score and CVD [48], and smoking and mortality [49]. Importantly, within the MIDUS cohort, GrimAge and DunedinPACE proved to be the most sensitive epigenome measures to explain the relationship between an individual’s sense of loneliness and their chronic health conditions [50]. Our findings showing the linkages to renal health add to the growing evidence that GrimAge and DunedinPACE may be more sensitive epigenetic indices than earlier epigenome clock measures for understanding the social determinants of health and age-related morbidity.

Our findings also concur with the other studies showing the predictive utility of epigenetic aging for understanding the progression to CKD. Accelerated epigenetic aging has previously been shown to be associated with an increased risk of CKD [15, 16]. Further, accelerated epigenetic aging was also found to be a robust marker for premature mortality among individuals with CKD [15]. Our study highlights the important roles of life course socioeconomic factors in shaping the epigenomic trajectories, including their association with renal aging. Specifically, our consideration of early life and adult SES factors underscores the significant influence of the accumulation of socioeconomic disadvantage across the life course on epigenetic aging [20]. Socioeconomic disadvantage early in life can summate across the life course and both initiate and shape aging trajectories, with more overt health consequences emerging during middle and older adulthood. Moreover, we showed that accelerated GrimAge and faster DunedinPACE pace of aging were robust predictors of larger decrements in eGFR across a decade. Although the participants in the MIDUS study were relatively healthy adults, type 2 diabetes and signs of atherosclerotic cardiovascular disease were prevalent, and faster decrements in eGFR would be significantly associated with a higher risk for CKD [17, 18].

Our exploratory analyses comparing NIH Black and white participants indicated that the mediation by epigenetic aging, on the association between life course SES and decrements in eGFR, was conditional on race, especially GrimAge. The moderated mediation analysis of the association between parental education and decrements in eGFR showed that the indirect effect through adult SES and GrimAge acceleration was significant only among the NH white participants but not in NH Black adults. Previous studies have also shown that the link between SES and epigenetic aging [20] or epigenetic aging and health outcomes [46] is weaker among Black adults relative to white adults. In fact, the earliest studies using the first-generation epigenetic clocks (i.e., Horvath and Hannum) indicated that Black individuals might experience slower aging than white adults [51]. It was only when the second-generation clocks (i.e., PhenoAge and GrimAge) and then the DunedinPACE indices became available that the findings shifted. Now, more recent studies typically conclude that Black relative to white adults tend to show faster GrimAge acceleration and DunedinPACE pace of aging [52]. While SES partially accounts for racial differences in accelerated aging between Black and white adults, other non-SES-related factors (e.g., frequent experiences of everyday discrimination) were not included in our analysis. When looking more comprehensively at historical and contemporary racial injustices, socioeconomic disadvantages were associated with accelerated epigenetic aging among NH Black adults [22]. Future studies should prioritize looking at the combined effect of SES and non-SES-related factors on epigenetic aging and the risk of CKD.

While this study successfully advanced the understanding of the mediating role of epigenetic aging in the association between life course SES and the risk of CKD, we should also acknowledge the limitations. First, parental education, a proxy for early life SES, was based on a retrospective recall that may be prone to bias. However, a prior analysis using data on siblings that were included in MIDUS showed a high concordance rate for reported parental education among the sibling pairs [53]. Our hypothetical model includes the possibility that the early programming of kidney function and regulation could also be impacted by adverse prenatal conditions and pregnancy outcomes, including premature birth, which can have persistent effects on nephron number and size. However, information on the fetal period and birthing is unavailable for the MIDUS participants who were recruited in adulthood. It should also be acknowledged that despite taking advantage of the longitudinal information acquired over decades of study and using a mediational statistical modeling approach, this was a correlational analysis, and causation cannot be definitively delineated. However, it is more parsimonious to consider that social and economic stressors are antecedent to the alterations in epigenetic aging clocks and that changes in the methylome and gene transcription contribute to why socioeconomic disadvantages are associated with faster age-related decrements in kidney function. These associations with renal health emerged even though the MIDUS participants were not selected based on kidney dysfunction. There were no individuals with stage 4 or 5 CKD, given that the inclusion criteria for this analysis included having eGFR above 60 mL/min/1.73 m^2^, which is often used as the diagnostic cutoff for renal disease. However, there were many individuals at risk for age-related decrements in kidney function, including those with signs of type 2 diabetes and hypertension. In addition, we had previously found that a period of hyperfiltration during middle age in NH Black adults could be a unique risk factor. Further, the evidence for race-related differences should be considered exploratory, given the small number of NH Black participants and the fact that most were recruited from a single city (Milwaukee, WI). Future studies are needed to replicate our analysis with a larger and more diverse sample of NH Black adults. Finally, it should be acknowledged that our measures of DNA methylation were obtained from cells in the blood, not from kidney tissue. In addition, we used whole blood, and some analyses have indicated that the individual variation in the leukocyte numbers and cell types in the blood can influence the amount of DNA methylation that is detected [54].

## Conclusion

Using a prospective cohort of middle-aged and older American adults, we have generated strong support for the primary hypothesis that DNA methylation indicators of accelerated epigenetic aging, especially GrimAge and the DunedinPACE pace of aging, statistically mediated the association between life course SES and age-related decrements in kidney function over 10 years. These findings affirm the sensitivity of epigenome indices to capture the cumulative negative aspects of socioeconomic disadvantage across the life course. Specifically, GrimAge and DunedinPACE evinced the most potential utility for identifying the clinical consequences of accelerated epigenetic aging because they were associated with the age-related decrements in glomerular filtration and renal clearance, a forerunner of more severe CKD. Our findings contribute to translational geroscience by highlighting the link between life course SES, an important social determinant of health and well-being, epigenetic aging, and the risk for age-related kidney dysfunction.

## Supplementary Information

Below is the link to the electronic supplementary material.

Supplementary file1 (DOCX 570 KB)

## Data Availability

Data is publicly available on the MIDUS Colectica Portal (https://midus.colectica.org/). The analyses in this study were not preregistered.
