# Supplementary material for: Epigenetic aging mediates the association between life course socioeconomic status and decrements in kidney function across a decade
Source: GeroScience. 2025 Jun 12;48(1):1241–57. doi: 10.1007/s11357-025-01728-0 (PMC12972445; doi:10.1007/s11357-025-01728-0)
Supplement: Supplementary file 1 — Supplementary file1 (DOCX 570 KB) [file 11357_2025_1728_MOESM1_ESM.docx]

**Supplementary Materials**

Epigenetic Aging Mediates the Association Between Life Course Socioeconomic Status and Decrements in Kidney Function Across a Decade

Agus Surachman^1,2^, Meera N. Harhay^1,3^, Rose Ann DiMaria-Ghalili^2^, Anthony S. Zannas^4^, David M. Almeida^5,6^, and Christopher L. Coe^7^

^1^Department of Epidemiology and Biostatistics, Dornsife School of Public Health, Drexel University, Philadelphia, PA, USA

^2^College of Nursing and Health Professions, Drexel University, Philadelphia, PA, USA

^3^Department of Medicine, College of Medicine, Drexel University, Philadelphia, PA, USA

^4^Department of Psychiatry, School of Medicine, University of North Carolina at Chapel Hill, Chapel Hill, NC, USA

^4^Department of Human Development and Family Studies, College of Health and Human Development, The Pennsylvania State University, University Park, PA, USA

^6^Center for Healthy Aging, The Pennsylvania State University, University Park, PA, USA

^5^Department of Psychology, University of Wisconsin-Madison, Madison, WI, USA

**Supplementary Tables**

- **Table S1**. Bivariate correlations among variables included in the current analysis
- **Table S2.** Full results from regression analysis on the association between life course SES and epigenetic-based age accelerations among all participants (*N* = 252)
- **Table S3.** Full results from regression analysis on the association between life course SES, epigenetic aging, and decrements in eGFR across a decade in all participants (*N* = 252)
- **Table S4.** Full results from mediation analysis on the mediating role of adult SES and DunedinPACE pace of aging on the association between parental education and decrements in eGFR across a decade (*N* = 252)
- **Table S5.** Full results from mediation analysis on the mediating role of adult SES and EAA GrimAge on the association between parental education and decrements in eGFR across a decade (*N* = 251)
- **Table S6.** Full results from regression analysis on the association between life course SES and epigenetic-based age accelerations in NH white participants (*N* = 190)
- **Table S7.** Full results from regression analysis on the association between life course SES and epigenetic-based age accelerations in NH Black participants (*N* = 62)
- **Table S8.** Full results from regression analysis on the association between life course SES, epigenetic aging, and decrements in eGFR across a decade in NH white participants (*N* = 190)
- **Table S9.** Full results from regression analysis on the association between life course SES, epigenetic aging, and decrements in eGFR across a decade in NH Black participants (*N* = 62)
- **Table S10.** Full results from moderated mediation analysis on the mediating role of adult SES and EAA GrimAge on the association between parental education and decrements in eGFR across a decade, and whether race moderated these mediations (*N* = 251)
- **Table S11.** Full results from moderated mediation analysis on the mediating role of adult SES and DunedinPACE on the association between parental education and decrements in eGFR across a decade, and whether race moderated these mediations (*N* = 251)

**Supplementary Figures**

- **Figure S1**. Hypothesized moderated mediation model of the mediating role of adult SES and epigenetic aging on the association between parental education and decrements in eGFR across a decade and whether race moderated these mediations.
- **Figure S2**. Results from the moderated mediation model of the mediating role of adult SES and EAA GrimAge on the association between parental education and decrements in eGFR across a decade and whether race moderated these mediations. Race moderated the association between EAA GrimAge and decrements in eGFR across a decade.

**Table S1.** Bivariate correlations among variables included in the current analysis (*N* = 252)

*Note*: Declines eGFR in mL/min/year/1.73 m^2^; Parental education: 0 = HS/GED or lower, 1 = some college or higher; adult SES: score; EAA = epigenetic age acceleration; age in years; follow-up in years; sex: 0 = male, 1 = female; race: 0 = NH white, 1 = NH Black; smoking: 0 = No, 1 = Yes; Obese: 0 = No, 1 = Yes; Elevated BP: 0 = No, 1 = Yes; Insulin Resistance: 0 = No, 1 = Yes.

**Table S2.** Full results from regression analysis on the association between life course SES and epigenetic-based age accelerations among all participants (*N* = 252)

|  | Outcome: Epigenetic Aging | | | | | |
| --- | --- | --- | --- | --- | --- | --- |
|  | Model 1 | | Model 2 | | Model 3 | |
|  | *B* (*SE*) | 95%*CI* | *B* (*SE*) | 95%*CI* | *B* (*SE*) | 95%*CI* |
| **EAA Horvath** |  |  |  |  |  |  |
| Intercept | 0.83 (1.56) | [-2.24, 3.90] | 1.22 (1.61) | [-1.95, 4.40] | 1.50 (1.80) | [-2.05, 5.05] |
| Parental education (0 = HS/GED or lower, 1 = higher) | 0.15 (0.52) | [-0.88, 1.18] | 0.28 (0.54) | [-0.78, 1.35] | 0.25 (0.55) | [-0.83, 1.34] |
| Age in MIDUS 2 (years) | -0.01 (0.03) | [-0.06, 0.05] | -0.00 (0.03) | [-0.06, 0.05] | -0.01 (0.03) | [-0.06, 0.05] |
| Sex (0 = male, 1 = female) | -0.89 (0.51) | [-1.89, 0.11] | -0.96 (0.51) | [-1.97, 0.05] | -0.95 (0.53) | [-1.99, 0.09] |
| Adult SES |  |  | -0.11 (0.11) | [-0.32, 0.11] | -0.11 (0.12) | [-0.34, 0.12] |
| Currently smoking |  |  |  |  | -0.28 (0.68) | [-1.62, 1.06] |
| Obese |  |  |  |  | -0.38 (0.54) | [-1.45, 0.69] |
| Elevated blood pressure |  |  |  |  | -0.04 (0.53) | [-1.09, 1.01] |
| Insulin resistance |  |  |  |  | 0.82 (0.70) | [-0.56, 2.20] |
| **Model Summary** | *R*^2^ = .01 | | *R*^2^ = .02 | | *R*^2^ = .02 | |
|  | *F* (3, 248) = 1.08 | | *F* (4, 247) = 1.04 | | *F* (8, 243) = 0.76 | |
| **EAA Horvath 2** |  |  |  |  |  |  |
| Intercept | -0.32 (1.14) | [-2.57, 1.92] | -0.48 (1.18) | [-2.81, 1.85] | 0.03 (1.32) | [-2.58, 2.64] |
| Parental education (0 = HS/GED, 1 = higher) | 0.09 (0.38) | [-0.66, 0.85] | 0.04 (0.40) | [-0.74, 0.82] | 0.01 (0.40) | [-0.79, 0.80] |
| Age in MIDUS 2 (years) | 0.00 (0.02) | [-0.04, 0.04] | 0.00 (0.02) | [-0.04, 0.04] | -0.00 (0.02) | [-0.04, 0.04] |
| Sex (0 = male, 1 = female) | 0.25 (0.37) | [-0.48, 0.98] | 0.28 (0.38) | [-0.46, 1.02] | 0.19 (0.39) | [-0.57, 0.96] |
| Adult SES |  |  | 0.04 (0.08) | [-0.12, 0.20] | 0.02 (0.09) | [-0.15, 0.19] |
| Currently smoking |  |  |  |  | -0.49 (0.50) | [-1.47, 0.50] |
| Obese |  |  |  |  | -0.04 (0.40) | [-0.83, 0.74] |
| Elevated blood pressure |  |  |  |  | 0.10 (0.39) | [-0.68, 0.87] |
| Insulin resistance |  |  |  |  | -0.07 (0.51) | [-1.08, 0.94] |
| **Model Summary** | *R*^2^ = .002 | | *R*^2^ = .003 | | *R*^2^ = .01 | |
|  | *F* (3, 248) = 0.17 | | *F* (4, 247) = 0.19 | | *F* (8, 243) = 0.23 | |
| **EAA Hannum** |  |  |  |  |  |  |
| Intercept | 1.37 (1.48) | [-1.55, 4.29] | 0.91 (1.53) | [-2.11, 3.93] | 1.64 (1.71) | [-1.73, 5.01] |
| Parental education (0 = HS/GED, 1 = higher) | -0.06 (0.50) | [-1.04, 0.92] | -0.22 (0.51) | [-1.23, 0.80] | -0.29 (0.52) | [-1.32, 0.75] |
| Age in MIDUS 2 (years) | -0.01 (0.03) | [-0.06, 0.04] | -0.02 (0.03) | [-0.07, 0.04] | -0.02 (0.03) | [-0.07, 0.03] |
| Sex (0 = male, 1 = female) | -1.28 (0.48) | [-2.23, -0.33] | -1.20 (0.49) | [-2.16, -0.24] | -1.33 (0.50) | [-2.32, -0.35] |
| Adult SES |  |  | 0.12 (0.10) | [-0.08, 0.33] | 0.08 (0.11) | [-0.14, 0.30] |
| Currently smoking |  |  |  |  | -0.54 (0.65) | [-1.82, 0.73] |
| Obese |  |  |  |  | 0.02 (0.52) | [-0.99, 1.04] |
| Elevated blood pressure |  |  |  |  | 0.12 (0.51) | [-0.88, 1.12] |
| Insulin resistance |  |  |  |  | -0.87 (0.66) | [-2.18, 0.43] |
| **Model Summary** | *R*^2^ = .03 | | *R*^2^ = .03 | | *R*^2^ = .04 | |
|  | *F* (3, 248) = 2.35 | | *F* (4, 247) = 2.11 | | *F* (8, 243) = 1.36 | |
| **EAA PhenoAge** |  |  |  |  |  |  |
| Intercept | 0.13 (2.29) | [-4.37, 4.63] | 1.11 (2.36) | [-3.54, 5.75] | 0.82 (2.62) | [-4.33, 5.98] |
| Parental education (0 = HS/GED, 1 = higher) | -0.09 (0.77) | [-1.60, 1.42] | 0.23 (0.79) | [-1.32, 1.79] | 0.42 (0.80) | [-1.16, 1.99] |
| Age in MIDUS 2 (years) | -0.00 (0.04) | [-0.08, 0.08] | 0.01 (0.04) | [-0.07, 0.08] | -0.01 (0.04) | [-0.09, 0.07] |
| Sex (0 = male, 1 = female) | -0.07 (0.74) | [-1.53, 1.39] | -0.24 (0.75) | [-1.71, 1.23] | -0.33 (0.77) | [-1.84, 1.17] |
| Adult SES |  |  | -0.26 (0.16) | [-0.57, 0.06] | -0.21 (0.17) | [-0.54, 0.13] |
| Currently smoking |  |  |  |  | 0.38 (0.99) | [-1.57, 2.33] |
| Obese |  |  |  |  | 0.23 (0.79) | [-1.32, 1.79] |
| Elevated blood pressure |  |  |  |  | 1.51 (0.78) | [-0.02, 3.04] |
| Insulin resistance |  |  |  |  | 0.60 (1.01) | [-1.40, 2.60] |
| **Model Summary** | *R*^2^ = .00 | | *R*^2^ = .01 | | *R*^2^ = .03 | |
|  | *F* (3, 248) = 0.01 | | *F* (4, 247) = 0.66 | | *F* (8, 243) = 0.96 | |
| **EAA GrimAge** |  |  |  |  |  |  |
| Intercept | 3.45 (1.99) | [-0.47, 7.37] | **6.41 (1.93)** | **[2.61, 10.21]** | -0.62 (1.70) | [-3.97, 2.73] |
| Parental education (0 = HS/GED, 1 = higher) | **-2.05 (0.67)** | **[-3.37, -0.74]** | -1.08 (0.65) | [-2.36, 0.19] | -0.37 (0.52) | [-1.39, 0.66] |
| Age in MIDUS 2 (years) | -0.03 (0.03) | [-0.10, 0.04] | -0.01 (0.03) | [-0.07, 0.06] | 0.03 (0.03) | [-0.03, 0.08] |
| Sex (0 = male, 1 = female) | **-2.09 (0.65)** | **[-3.37, -0.82]** | **-2.59 (0.61)** | **[-3.80, -1.38]** | **-1.61 (0.50)** | **[-2.59, -0.64]** |
| Adult SES |  |  | **-0.78 (0.13)** | **[-1.03, -0.52]** | **-0.40 (0.11)** | **[-0.62, -0.18]** |
| Currently smoking |  |  |  |  | **7.74 (0.64)** | **[6.48, 9.01]** |
| Obese |  |  |  |  | 0.55 (0.51) | [-0.46, 1.56] |
| Elevated blood pressure |  |  |  |  | 1.51 (0.51) | [0.52, 2.51] |
| Insulin resistance |  |  |  |  | 0.48 (0.67) | [-0.83, 1.80] |
|  | *R*^2^ = .07 | | *R*^2^ = .19 | | *R*^2^ = .50 | |
|  | ***F* (3, 247) = 6.34** | | ***F* (4, 246) = 14.21** | | ***F* (8, 242) = 30.13** | |
| **DunedinPACE Pace of Aging** |  |  |  |  |  |  |
| Intercept | **1.10 (0.05)** | **[1.00, 1.20]** | **1.17 (0.05)** | **[1.08, 1.27]** | **1.03 (0.05)** | **[0.93, 1.12]** |
| Parental education (0 = HS/GED, 1 = higher) | **-0.07 (0.02)** | **[-0.10, -0.04]** | **-0.05 (0.02)** | **[-0.08, -0.01]** | -0.02 (0.02) | [-0.05, 0.01] |
| Age in MIDUS 2 (years) | -0.00 (0.00) | [-0.00, 0.00] | -0.00 (0.00) | [-0.00, 0.00] | 0.00 (0.00) | [-0.00, 0.00] |
| Sex (0 = male, 1 = female) | -0.02 (0.02) | [-0.05, 0.01] | **-0.03 (0.02)** | **[-0.07, -0.00]** | -0.02 (0.01) | [-0.05, 0.01] |
| Adult SES |  |  | **-0.02 (0.00)** | **[-0.03, -0.01]** | **-0.01 (0.00)** | **[-0.02, -0.00]** |
| Currently smoking |  |  |  |  | **0.11 (0.02)** | **[0.08, 0.15]** |
| Obese |  |  |  |  | **0.06 (0.02)** | **[0.03, 0.09]** |
| Elevated blood pressure |  |  |  |  | **0.05 (0.01)** | **[0.02, 0.07]** |
| Insulin resistance |  |  |  |  | **0.04 (0.02)** | **[0.00, 0.08]** |
| **Model Summary** | *R*^2^ =.07 | | *R*^2^ = .18 | | *R*^2^ = .37 | |
|  | ***F* (3, 248) = 6.16** | | ***F* (4, 247) = 13.48** | | ***F* (8, 243) = 17.52** | |

*Note*: *B* = unstandardized regression coefficient, *SE* = standard error; *CI* = confidence intervals; EAA = epigenetic age acceleration. Bolded numbers indicate significant association (*p* < .05).

**Table S3.** Full results from regression analysis on the association between life course SES, epigenetic aging, and decrements in eGFR across a decade among all participants (*N* = 252)

|  | Outcome: Decrements in eGFR Across a Decade | | | | | |
| --- | --- | --- | --- | --- | --- | --- |
|  | Model 1 | | Model 2 | | Model 3 | |
|  | *B* (*SE*) | 95%*CI* | *B* (*SE*) | 95%*CI* | *B* (*SE*) | 95%*CI* |
| Intercept | **-22.16 (10.28)** | **[-42.41, -1.90]** | 16.05 (10.28) | [-36.29, 4.20] | 14.76 (10.47) | [-35.38, 5.87] |
| Parental education (0 = HS/GED, 1 = higher) | **-5.75 (2.03)** | **[-9.76, -1.74]** | -3.62 (2.08) | [-7.71, 0.48] | -3.24 (2.07) | [-7.32, 0.83] |
| Age in MIDUS 2 (years) | 0.03 (0.10) | [-0.18, 0.23] | 0.06 (0.10) | [-0.14, 0.26] | 0.04 (0.11) | [-0.16, 0.25] |
| Sex (0 = male, 1 = female) | 3.69 (1.94) | [-0.14, 7.52] | 3.67 (2.00) | [-0.26, 7.60] | 3.86 (1.99) | [-0.06, 7.77] |
| Follow-up (years) | **2.90 (0.77)** | **[1.39, 4.41]** | **2.68 (0.76)** | **[1.19, 4.17]** | **2.60 (0.75)** | **[1.13, 4.07]** |
| Adult SES |  |  | **-1.13 (0.44)** | **[-1.99, -0.26]** | **-0.97 (0.45)** | **[-1.84, -0.09]** |
| EAA GrimAge |  |  | 0.25 (0.20) | [-0.15, 0.64] | **0.62 (0.25)** | **[0.12, 1.12]** |
| Currently smoking |  |  |  |  | **-7.50 (3.18)** | **[-13.76, -1.25]** |
| Obese |  |  |  |  | **4.17 (2.01)** | **[0.21, 8.12]** |
| Elevated blood pressure |  |  |  |  | -2.59 (2.01) | [-6.55, 1.38] |
| Insulin resistance |  |  |  |  | 1.34 (2.61) | [-3.80, 6.48] |
| **Model Summary** | *R*^2^ =.09 | | *R*^2^ =.13 | | *R*^2^ =.17 | |
|  | ***F* (4, 247) = 5.99** | | ***F* (6, 244) = 6.12** | | ***F* (10, 240) = 4.98** | |
| Intercept | **-22.16 (10.28)** | **[-42.41, -1.90]** | -36.69 (13.25) | [-62.78, -10.59] | **-40.12 (13.49)** | **[-66.70, -13.55]** |
| Parental education (0 = HS/GED, 1 = higher) | **-5.75 (2.03)** | **[-9.76, -1.74]** | -3.14 (2.08) | [-7.24, 0.96] | -3.07 (2.08) | [-7.16, 1.02] |
| Age in MIDUS 2 (years) | 0.03 (0.10) | [-0.18, 0.23] | 0.08 (0.10) | [-0.12, 0.28] | 0.08 (0.11) | [-0.13, 0.29] |
| Sex (0 = male, 1 = female) | 3.69 (1.94) | [-0.14, 7.52] | 3.54 (1.93) | [-0.26, 7.34] | 3.25 (1.95) | [-0.59, 7.09] |
| Follow-up (years) | **2.90 (0.77)** | **[1.39, 4.41]** | **2.62 (0.75)** | **[1.14, 4.10]** | **2.64 (0.74)** | **[1.18, 4.11]** |
| Adult SES |  |  | **-0.93 (0.43)** | **[-1.79, -0.08]** | **-0.95 (0.44)** | **[-1.82, -0.08]** |
| DunedinPACE |  |  | **19.27 (7.87)** | **[3.77, 34.77]** | **23.60 (8.85)** | **[6.16, 41.03]** |
| Currently smoking |  |  |  |  | **-5.50 (2.71)** | **[-10.83, -0.17]** |
| Obese |  |  |  |  | 2.91 (2.08) | [-1.18, 7.00] |
| Elevated blood pressure |  |  |  |  | -2.93 (2.01) | [-6.88, 1.02] |
| Insulin resistance |  |  |  |  | 1.24 (2.60) | [-3.88, 6.36] |
| **Model Summary** | *R*^2^ =.09 | | *R*^2^ =.14 | | *R*^2^ =.17 | |
|  | ***F* (4, 247) = 5.99** | | ***F* (6, 245) = 6.89** | | ***F* (10, 241) = 5.08** | |

*Note*: *B* = unstandardized regression coefficient, *SE* = standard error; *CI* = confidence intervals; EAA = epigenetic age acceleration. Bolded numbers indicate significant association (*p* < .05).

**Table S4.** Full results from mediation analysis on the mediating role of adult SES and DunedinPACE pace of aging on the association between parental education and decrements in eGFR across a decade (*N* = 252)

|  | Adult SES | | DunedinPACE | | Declines in eGFR | | |
| --- | --- | --- | --- | --- | --- | --- | --- |
|  | *B* (*SE*) | 95%*CI* | *B* (*SE*) | 95%*CI* | *B* (*SE*) | | 95%*CI* |
| Constant | **6.07 (1.50)** | **[3.12, 9.01]** | **0.97 (0.08)** | **[0.82, 1.12]** | **-40.12 (13.49)** | | **[-66.70, -13.55]** |
| Parental education | **0.83 (0.30)** | **[0.23, 1.42]** | -0.03 (0.02) | [-0.06, 0.00] | -3.07 (2.08) | | [-7.16, 1.02] |
| Adult SES |  |  | **-0.01 (0.00)** | **[-0.02, -0.00]** | **-0.95 (0.44)** | | **[-1.82, -0.08]** |
| DunedinPACE2 |  |  |  |  | **23.60 (8.85)** | | **[6.16, 41.03]** |
| Age | 0.01 (0.02) | [-0.02, 0.04] | -0.00 (0.00) | [-0.00, 0.00] | 0.08 (0.10) | | [-0.13, 0.29] |
| Sex | **-0.83 (0.28)** | **[-1.38, -0.27]** | -0.02 (0.01) | [-0.05, 0.01] | 3.25 (1.95) | | [-0.59, 7.09] |
| Follow-up | -0.04 (0.11) | [-0.26, 0.18] | 0.01 (0.01) | [-0.01, 0.02] | **2.64 (0.74)** | | **[1.18, 4.11]** |
| Smoking | **-1.58 (0.36)** | **[-2.29, -0.87]** | **0.11 (0.02)** | **[0.08, 0.15]** | **-5.50 (2.70)** | | **[-10.83, -0.17]** |
| Obese | **-0.99 (0.29)** | **[-1.56, -0.42]** | **0.06 (0.01)** | **[0.03, 0.09]** | 2.91 (2.08) | | [-1.18, 7.00] |
| Elevated blood pressure | 0.04 (0.29) | [-0.54, 0.61] | **0.04 (0.01)** | **[0.02, 0.07]** | -2.93 (2.01) | | [-6.88, 1.02] |
| Insulin resistance | **-0.86 (0.38)** | **[-1.61, -0.12]** | **0.04 (0.02)** | **[0.00, 0.08]** | 1.24 (2.60) | | [-3.87, 6.36] |
| **Model Summary** | *R*^2^ = .21 | | *R*^2^ = .37 | | *R*^2^ = .17 | | |
|  | ***F* (8, 243) = 8.24** | | ***F* (9, 242) = 15.69** | | ***F* (10, 241) = 5.08** | | |
| **Indirect Effects** |  | | **Effect (boot. *SE*)** | | **95%*CI* (bootstrapped)** | | |
| Parental education 🡪 Adult SES 🡪 Decrements in eGFR | | | **-0.79 (0.45)** |  | **[-1.79, -0.05]** |  | |
| Parental education 🡪 DunedinPACE 🡪 Decrements in eGFR | | | -0.64 (0.47) |  | [-1.75, 0.04] |  | |
| Parental education 🡪 Adult SES 🡪 DunedinPACE 🡪 Decrements in eGFR | | | **-0.20 (0.14)** |  | **[-0.55, -0.02]** |  | |
| Total indirect effects | | | **-1.62 (0.74)** |  | **[-3.26, -0.39]** |  | |

*Note*: *B* = unstandardized regression coefficient, *SE* = standard error; *boot. SE* = bootstrapped standard error, *CI* = confidence interval, EAA = epigenetic age acceleration. Bolded numbers indicate significant association (*p* < .05).

**Table S5.** Full results from mediation analysis on the mediating role of adult SES and EAA GrimAge on the association between parental education and decrements in eGFR across a decade (*N* = 251)

|  | Adult SES | | EAA GrimAge | | Decrements in eGFR | | |
| --- | --- | --- | --- | --- | --- | --- | --- |
|  | *B* (*SE*) | 95%*CI* | *B* (*SE*) | 95%*CI* | *B* (*SE*) | | 95%*CI* |
| Constant | **6.09 (1.50)** | **[3.14, 9.04]** | -3.36 (2.67) | [-8.62, 1.90] | -14.76 (10.47) | | [-35.38, 5.87] |
| Parental education | **0.84 (0.30)** | **[0.24, 1.43]** | -0.50 (0.53) | [-1.54, 0.54] | -3.24 (2.07) | | [-7.32, 0.83] |
| Adult SES |  |  | **-0.40 (0.11)** | **[-0.61, -0.18]** | **-0.97 (0.44)** | | **[-1.84, -0.09]** |
| EAA GrimAge |  |  |  |  | **0.62 (0.25)** | | **[0.13, 1.12]** |
| Age | 0.01 (0.02) | [-0.02, 0.04] | 0.03 (0.03) | [-0.03, 0.08] | 0.04 (0.11) | | [-0.16, 0.25] |
| Sex | **-0.82 (0.28)** | **[-1.37, -0.26]** | **-1.64 (0.50)** | **[-2.62, -0.66]** | 3.86 (1.99) | | [-0.06, 7.77] |
| Follow-up | -0.04 (0.11) | [-0.26, 0.18] | 0.25 (0.19) | [-0.12, 0.63] | 2.60 (0.75) | | [-1.13, 4.07] |
| Smoking | **-1.57 (0.36)** | **[-2.28, -0.86]** | **7.71 (0.64)** | **[6.44, 8.98]** | **-7.50 (3.18)** | | **[-13.76, -1.25]** |
| Obese | **-0.98 (0.29)** | **[-1.55, -0.41]** | 0.54 (0.51) | [-0.47, 1.55] | **4.17 (2.01)** | | **[0.21, 8.12]** |
| Elevated blood pressure | 0.06 (0.29) | [-0.52, 0.63] | **1.48 (0.51)** | **[0.49, 2.48]** | -2.59 (2.01) | | [-6.55, 1.38] |
| Insulin resistance | **-0.90 (0.38)** | **[-1.66, -0.15]** | 0.43 (0.67) | [-0.88, 1.75] | 1.34 (2.61) | | [-3.80, 6.48] |
| **Model Summary** | *R*^2^ = .21 | | *R*^2^ = .50 | | *R*^2^ = .17 | | |
|  | ***F* (8, 242) = 8.20** | | ***F* (9, 241) = 27.07** | | ***F* (10, 240) = 4.98** | | |
| **Indirect Effects** |  | | **Effect (boot. *SE*)** | | **95%*CI* (bootstrapped)** | | |
| Parental education 🡪 adult SES 🡪 Decrements in eGFR | | | **-0.81 (0.44)** |  | **[-1.77, -0.06]** |  | |
| Parental education 🡪 EAA GrimAge 🡪 Decrements in eGFR | | | -0.31 (0.38) |  | [-1.23, 0.28] |  | |
| Parental education 🡪 adult SES 🡪 EAA GrimAge 🡪 Decrements in eGFR | | | -0.21 (0.17) |  | [-0.62, 0.00] |  | |
| Total indirect effects | | | **-1.33 (0.67)** |  | **[-2.84, -0.22]** |  | |

*Note*: *B* = unstandardized regression coefficient, *SE* = standard error; *boot. SE* = bootstrapped standard error, *CI* = confidence interval, EAA = epigenetic age acceleration. Bolded numbers indicate significant association (*p* < .05).

**Table S6.** Full results from regression analysis on the association between life course SES and epigenetic-based age accelerations in NH white participants (*N* = 190)

|  | Outcome: Epigenetic Aging | | | | | |
| --- | --- | --- | --- | --- | --- | --- |
|  | Model 1 | | Model 2 | | Model 3 | |
|  | *B* (*SE*) | 95%*CI* | *B* (*SE*) | 95%*CI* | *B* (*SE*) | 95%*CI* |
| **EAA GrimAge** |  |  |  |  |  |  |
| Intercept | -0.40 (2.11) | [-4.56, 3.76] | 3.02 (2.29) | [-1.48, 7.53] | -2.93 (1.99) | [-6.85, 0.99] |
| Parental education (0 = HS/GED, 1 = higher) | -1.31 (0.69) | [-2.67, 0.04] | -0.80 (0.68) | [-2.15, 0.55] | 0.16 (0.54) | [-0.91, 1.23] |
| Age in MIDUS 2 (years) | 0.02 (0.04) | [-0.05, 0.10] | 0.01 (0.04) | [-0.06, 0.08] | 0.05 (0.03) | [-0.01, 0.10] |
| Sex (0 = male, 1 = female) | **-1.57 (0.68)** | **[-2.92, -0.22]** | **-2.02 (0.68)** | **[-3.36, -0.68]** | **-1.49 (0.54)** | **[-2.56, -0.42]** |
| Adult SES |  |  | **-0.54 (0.16)** | **[-0.85, -0.23]** | **-0.29 (0.13)** | **[-0.55, -0.03]** |
| Currently smoking |  |  |  |  | **8.21 (0.74)** | **[6.75, 9.68]** |
| Obese |  |  |  |  | 0.87 (0.58) | [-0.27, 2.00] |
| Elevated blood pressure |  |  |  |  | **1.13 (0.56)** | **[0.02, 2.24]** |
| Insulin resistance |  |  |  |  | 0.42 (0.90) | [-1.36, 2.19] |
| **Model Summary** | *R*^2^ = .05 | | *R*^2^ = .11 | | *R*^2^ = .47 | |
|  | ***F* (3, 185) = 3.37** | | ***F* (4, 184) = 5.54** | | ***F* (8, 180) = 19.94** | |
| **DunedinPACE Pace of Aging** |  |  |  |  |  |  |
| Intercept | **0.98 (0.05)** | **[0.89, 1.08]** | **1.05 (0.05)** | **[0.95, 1.16]** | **0.94 (0.06)** | **[0.83, 1.05]** |
| Parental education (0 = HS/GED, 1 = higher) | **-0.04 (0.02)** | **[-0.07, -0.01]** | **-0.03 (0.02)** | **[-0.06, 0.00]** | -0.02 (0.02) | [-0.05, 0.01] |
| Age in MIDUS 2 (years) | 0.00 (0.00) | [-0.00, 0.00] | 0.00 (0.00) | [-0.00, 0.00] | 0.00 (0.00) | [-0.00, 0.00] |
| Sex (0 = male, 1 = female) | -0.02 (0.02) | [-0.05, 0.01] | -0.03 (0.02) | [-0.06, 0.00] | -0.02 (0.02) | [-0.05, 0.01] |
| Adult SES |  |  | **-0.01 (0.00)** | **[-0.02, -0.00]** | -0.01 (0.00) | [-0.01, 0.00] |
| Currently smoking |  |  |  |  | **0.09 (0.02)** | **[0.05, 0.13]** |
| Obese |  |  |  |  | **0.07 (0.02)** | **[0.04, 0.10]** |
| Elevated blood pressure |  |  |  |  | 0.02 (0.02) | [-0.01, 0.05] |
| Insulin resistance |  |  |  |  | 0.04 (0.02) | [-0.01, 0.09] |
| **Model Summary** | *R*^2^ =.05 | | *R*^2^ = .09 | | *R*^2^ = .24 | |
|  | ***F* (3, 186) = 2.99** | | ***F* (4, 185) = 4.42** | | ***F* (8, 181) = 7.28** | |

*Note*: *B* = unstandardized regression coefficient, *SE* = standard error; *CI* = confidence intervals; EAA = epigenetic age acceleration. Bolded numbers indicate significant association (*p* < .05).

**Table S7.** Full results from regression analysis on the association between life course SES and epigenetic-based age accelerations in NH Black participants (*N* = 62)

|  | Outcome: Epigenetic Aging | | | | | |
| --- | --- | --- | --- | --- | --- | --- |
|  | Model 1 | | Model 2 | | Model 3 | |
|  | *B* (*SE*) | 95%*CI* | *B* (*SE*) | 95%*CI* | *B* (*SE*) | 95%*CI* |
| **EAA GrimAge** |  |  |  |  |  |  |
| Intercept | **9.65 (4.56)** | **[0.52, 18.78]** | **9.41 (4.41)** | **[0.59, 18.23]** | 3.02 (4.00) | [-5.00, 11.03] |
| Parental education (0 = HS/GED, 1 = higher) | -2.59 (1.73) | [-6.05, 0.88] | -2.06 (1.69) | [-5.44, 1.32] | -2.33 (1.49) | [-5.31, 0.65] |
| Age in MIDUS 2 (years) | -0.08 (0.09) | [-0.25, 0.09] | -0.02 (0.09) | [-0.20, 0.15] | -0.02 (0.08) | [-0.17, 0.14] |
| Sex (0 = male, 1 = female) | **-3.97 (1.43)** | **[-6.83, -1.11]** | **-3.96 (1.38)** | **[-6.73, -1.20]** | -1.87 (1.29) | [-4.46, -0.72] |
| Adult SES |  |  | **-0.77 (0.34)** | **[-1.45, -0.09]** | -0.25 (0.31) | [-0.86, 0.37] |
| Currently smoking |  |  |  |  | **6.96 (1.41)** | **[4.14, 9.78]** |
| Obese |  |  |  |  | -0.47 (1.22) | [-2.93, 1.98] |
| Elevated blood pressure |  |  |  |  | 1.86 (1.25) | [-0.65, 4.36] |
| Insulin resistance |  |  |  |  | -0.04 (1.22) | [-2.49, 2.41] |
| **Model Summary** | *R*^2^ = .14 | | *R*^2^ = .22 | | *R*^2^ = .48 | |
|  | ***F* (3, 58) = 3.25** | | ***F* (4, 57) = 3.91** | | ***F* (8, 53) = 6.01** | |
| **DunedinPACE Pace of Aging** |  |  |  |  |  |  |
| Intercept | **1.26 (0.11)** | **[1.03, 1.49]** | **1.26 (0.11)** | **[1.03, 1.49]** | **1.09 (0.10)** | **[0.88, 1.30]** |
| Parental education (0 = HS/GED, 1 = higher) | -0.08 (0.04) | [-0.17, 0.01] | -0.07 (0.04) | [-0.16, 0.02] | -0.07 (0.04) | [-0.14, 0.01] |
| Age in MIDUS 2 (years) | -0.00 (0.00) | [-0.01, 0.00] | -0.00 (0.00) | [-0.01, 0.00] | -0.00 (0.00) | [-0.01, 0.00] |
| Sex (0 = male, 1 = female) | -0.04 (0.04) | [-0.11, 0.03] | -0.04 (0.04) | [-0.11, 0.03] | 0.00 (0.03) | [-0.07, 0.07] |
| Adult SES |  |  | -0.01 (0.01) | [-0.03, 0.01] | 0.00 (0.01) | [-0.01, 0.02] |
| Currently smoking |  |  |  |  | **0.17 (0.04)** | **[0.10, 0.24]** |
| Obese |  |  |  |  | 0.02 (0.03) | [-0.04, 0.09] |
| Elevated blood pressure |  |  |  |  | 0.06 (0.03) | [-0.01, 0.12] |
| Insulin resistance |  |  |  |  | 0.01 (0.03) | [-0.06, 0.07] |
| **Model Summary** | *R*^2^ =.08 | | *R*^2^ = .09 | | *R*^2^ = .39 | |
|  | *F* (3, 58) = 1.62 | | *F* (4, 57) = 1.47 | | ***F* (8, 53) = 4.27** | |

*Note*: *B* = unstandardized regression coefficient, *SE* = standard error; *CI* = confidence intervals; EAA = epigenetic age acceleration. Bolded numbers indicate significant association (*p* < .05).

**Table S8.** Full results from regression analysis on the association between life course SES, epigenetic aging, and decrements in eGFR across a decade in NH white participants (*N* = 190)

|  | Outcome: Changes in eGFR Across a Decade | | | | | |
| --- | --- | --- | --- | --- | --- | --- |
|  | Model 1 | | Model 2 | | Model 3 | |
|  | *B* (*SE*) | 95%*CI* | *B* (*SE*) | 95%*CI* | *B* (*SE*) | 95%*CI* |
| Intercept | **-20.62 (10.12)** | **[-40.57, -0.66]** | -9.75 (10.22) | [-29.92, 10.41] | 13.24 (10.43) | [-33.83, 7.34] |
| Parental education (0 = HS/GED, 1 = higher) | **-4.94 (1.98)** | **[-8.84, -1.04]** | -2.88 (1.97) | [-6.76, 0.99] | -3.30 (1.94) | [-7.13, 0.54] |
| Age in MIDUS 2 (years) | 0.08 (0.10) | [-1.12, 0.28] | 0.05 (0.10) | [-0.14, 0.25] | 0.07 (0.10) | [-0.13, 0.27] |
| Sex (0 = male, 1 = female) | 3.55 (1.92) | [-0.24, 7.34] | **3.92 (1.93)** | **[0.11, 7.73]** | **4.93 (1.92)** | **[1.14, 8.73]** |
| Follow-up (years) | **2.41 (0.75)** | **[0.92, 3.89]** | **1.97 (0.73)** | **[0.53, 3.42]** | **2.14 (0.72)** | **[0.73, 3.56]** |
| Adult SES |  |  | -0.90 (0.46) | [-1.80, 0.00] | -0.56 (0.47) | [-1.48, 0.36] |
| EAA GrimAge |  |  | **0.60 (0.21)** | **[0.19, 1.01]** | **0.99 (0.26)** | **[0.48, 1.51]** |
| Currently smoking |  |  |  |  | **-7.75 (3.36)** | **[-14.39, -1.12]** |
| Obese |  |  |  |  | **4.20 (2.02)** | **[0.22, 8.18]** |
| Elevated blood pressure |  |  |  |  | -3.85 (1.98) | [-7.76, 0.06] |
| Insulin resistance |  |  |  |  | 1.22 (3.14) | [-4.97, 7.42] |
| **Model Summary** | *R*^2^ =.09 | | *R*^2^ =.16 | | *R*^2^ =.22 | |
|  | ***F* (4, 185) = 4.31** | | ***F* (6, 182) = 5.73** | | ***F* (10, 178) = 4.92** | |
| Intercept | **-20.62 (10.12)** | **[-40.57, -0.66]** | **-43.42 (12.93)** | **[-68.94, -17.91]** | **-48.71 (13.21)** | **[-74.77, -22.64]** |
| Parental education (0 = HS/GED, 1 = higher) | **-4.94 (1.98)** | **[-8.84, -1.04]** | -2.52 (1.95) | [-6.37, 1.34] | -2.79 (1.97) | [-6.67, 1.09] |
| Age in MIDUS 2 (years) | 0.08 (0.10) | [-1.12, 0.28] | 0.07 (0.10) | [-0.12, 0.26] | 0.11 (0.10) | [-0.09, 0.31] |
| Sex (0 = male, 1 = female) | 3.55 (1.92) | [-0.24, 7.34] | 3.48 (1.88) | [-0.23, 7.19] | **4.01 (1.91)** | **[0.25, 7.77]** |
| Follow-up (years) | **2.41 (0.75)** | **[0.92, 3.89]** | **2.00 (0.72)** | **[0.58, 3.42]** | **2.17 (0.72)** | **[0.74, 3.60]** |
| Adult SES |  |  | -0.84 (0.45) | [-1.72, 0.05] | -0.65 (0.47) | [-1.57, 0.27] |
| DunedinPACE |  |  | **33.09 (8.64)** | **[16.04, 50.14]** | **33.75 (9.41)** | **[15.19, 52.31]** |
| Currently smoking |  |  |  |  | -2.80 (2.75) | [-8.24, 2.63] |
| Obese |  |  |  |  | 2.64 (2.13) | [-1.56, 6.84] |
| Elevated blood pressure |  |  |  |  | -3.82 (1.98) | [-7.72, 0.08] |
| Insulin resistance |  |  |  |  | 1.39 (3.10) | [-4.74, 7.52] |
| **Model Summary** | *R*^2^ =.09 | | *R*^2^ =.18 | | *R*^2^ =.21 | |
|  | ***F* (4, 185) = 4.31** | | ***F* (6, 183) = 6.85** | | ***F* (10, 179) = 4.74** | |

*Note*: *B* = unstandardized regression coefficient, *SE* = standard error; *CI* = confidence intervals; EAA = epigenetic age acceleration. Bolded numbers indicate significant association (*p* < .05).

**Table S9.** Full results from regression analysis on the association between life course SES, epigenetic aging, and decrements in eGFR across a decade in NH Black participants (*N* = 62)

|  | Outcome: Changes in eGFR Across a Decade | | | | | |
| --- | --- | --- | --- | --- | --- | --- |
|  | Model 1 | | Model 2 | | Model 3 | |
|  | *B* (*SE*) | 95%*CI* | *B* (*SE*) | 95%*CI* | *B* (*SE*) | 95%*CI* |
| Intercept | -27.49 (30.12) | [-87.81, 32.82] | -21.99 (31.26) | [-84.63, 40.65] | -13.39 (33.03) | [-79.69, 52.91] |
| Parental education (0 = HS/GED, 1 = higher) | -4.87 (6.61) | [-18.10, 8.37] | -5.01 (6.76) | [-18.56, 8.55] | -1.85 (7.24) | [-16.39, 12.69] |
| Age in MIDUS 2 (years) | -0.05 (0.32) | [-0.69, 0.60] | 0.03 (0.34) | [-0.64, 0.71] | -0.04 (0.36) | [-0.75, 0.68] |
| Sex (0 = male, 1 = female) | 3.33 (5.57) | [-7.83, 14.49] | 1.45 (5.91) | [-10.40, 13.30] | -0.81 (6.28) | [-13.42, 11.81] |
| Follow-up (years) | 3.98 (2.31) | [-0.64, 8.61] | 3.87 (2.34) | [-0.82, 8.55] | 3.35 (2.43) | [-1.53, 8.23] |
| Adult SES |  |  | -1.63 (1.40) | [-4.43, 1.17] | -1.93 (1.46) | [-4.86, 1.00] |
| EAA GrimAge |  |  | -0.49 (0.53) | [-1.55, 0.56] | -0.17 (0.65) | [-1.48, 1.14] |
| Currently smoking |  |  |  |  | -8.36 (8.18) | [-24.79, 8.08] |
| Obese |  |  |  |  | 4.71 (5.85) | [-7.04, 16.46] |
| Elevated blood pressure |  |  |  |  | 1.67 (6.05) | [-10.47, 13.81] |
| Insulin resistance |  |  |  |  | 1.42 (5.81) | [-10.25, 13.08] |
| **Model Summary** | *R*^2^ =.07 | | *R*^2^ =.10 | | *R*^2^ = .14 | |
|  | *F* (4, 57) = 1.12 | | *F* (6, 55) = 1.04 | | *F* (10, 51) = 0.80 | |
| Intercept | -27.49 (30.12) | [-87.81, 32.82] | -26.56 (41.89) | [-110.52, 57.39] | -28.13 (43.05) | [-114.55, 58.29] |
| Parental education (0 = HS/GED, 1 = higher) | -4.87 (6.61) | [-18.10, 8.37] | -4.18 (6.89) | [-17.98, 9.62] | -0.60 (7.26) | [-15.18, 13.97] |
| Age in MIDUS 2 (years) | -0.05 (0.32) | [-0.69, 0.60] | 0.04 (0.34) | [-0.65, 0.73] | -0.01 (0.36) | [-0.73, 0.71] |
| Sex (0 = male, 1 = female) | 3.33 (5.57) | [-7.83, 14.49] | 3.20 (5.67) | [-8.17, 14.57] | -0.52 (6.15) | [-12.87, 8.24] |
| Follow-up (years) | 3.98 (2.31) | [-0.64, 8.61] | 4.09 (2.35) | [-0.62, 8.81] | 3.37 (2.43) | [-1.51, 8.24] |
| Adult SES |  |  | -1.27 (1.36) | [-4.01, 1.46] | -1.93 (1.45) | [-4.85, 0.98] |
| DunedinPACE |  |  | -2.00 (20.49) | [-43.07, 39.07] | 12.85 (25.06) | [-37.45, 63.15] |
| Currently smoking |  |  |  |  | -11.70 (7.99) | [-27.73, 4.34] |
| Obese |  |  |  |  | 4.51 (5.86) | [-7.25, 16.28] |
| Elevated blood pressure |  |  |  |  | 0.61 (6.09) | [-11.62, 12.83] |
| Insulin resistance |  |  |  |  | 1.32 (5.80) | [-10.33, 12.97] |
| **Model Summary** | *R*^2^ =.07 | | *R*^2^ = .09 | | *R*^2^ = .14 | |
|  | *F* (4, 57) = 1.12 | | *F* (6, 55) = 0.88 | | *F* (10, 51) = 0.83 | |

*Note*: *B* = unstandardized regression coefficient, *SE* = standard error; *CI* = confidence intervals; EAA = epigenetic age acceleration. Bolded numbers indicate significant association (*p* < .05).

**Table S10.** Full results from moderated mediation analysis on the mediating role of adult SES and EAA GrimAge on the association between parental education and decrements in eGFR across a decade, and whether race moderated these mediations (*N* = 251)

|  | Adult SES | | EAA GrimAge | | Changes in eGFR | | |
| --- | --- | --- | --- | --- | --- | --- | --- |
|  | *B* (*SE*) | 95%*CI* | *B* (*SE*) | 95%*CI* | *B* (*SE*) | | 95%*CI* |
| Constant | 0.77 (1.50) | [-2.18, 3.72] | **-5.47 (2.58)** | **[-10.55, -0.38]** | -16.56 (10.34) | | [-36.93, 3.81] |
| Parental education | **0.84 (0.30)** | **[0.24, 1.43]** | -0.50 (0.53) | [-1.54, 0.54] | -3.14 (2.06) | | [-7.20, 0.92] |
| Adult SES |  |  | **-0.40 (0.11)** | **[-0.61, -0.18]** | **-0.67 (0.54)** | | **[-1.73, 0.39]** |
| EAA GrimAge |  |  |  |  | **0.95 (0.29)** | | **[0.39, 1.51]** |
| Race |  |  |  |  | 0.44 (2.92) | | [-5.32, 6.19] |
| Adult SES*Race |  |  |  |  | -1.21 (1.09) | | [-3.36, 0.93] |
| EAA GrimAge*Race |  |  |  |  | **-1.03 (0.42)** | | **[-1.85, -0.22]** |
| Age | 0.01 (0.02) | [-0.02, 0.04] | 0.03 (0.03) | [-0.03, 0.08] | 0.04 (0.11) | | [-0.17, 0.25] |
| Sex | **-0.82 (0.28)** | **[-1.37, -0.26]** | **-1.64 (0.50)** | **[-2.62, -0.66]** | 3.78 (1.98) | | [-0.13, 7.69] |
| Follow-up | -0.04 (0.11) | [-0.26, 0.18] | 0.25 (0.19) | [-0.12, 0.63] | 2.33 (0.75) | | [0.85, 3.81] |
| Smoking | **-1.57 (0.36)** | **[-2.28, -0.86]** | **7.71 (0.64)** | **[6.44, 8.98]** | **-7.63 (3.16)** | | **[-13.86, -1.40]** |
| Obese | **-0.98 (0.29)** | **[-1.55, -0.41]** | 0.54 (0.51) | [-0.47, 1.55] | **4.21 (2.01)** | | **[0.25, 8.17]** |
| Elevated blood pressure | 0.06 (0.29) | [-0.52, 0.63] | **1.48 (0.51)** | **[0.49, 2.48]** | -2.58 (2.04) | | [-6.61, 1.45] |
| Insulin resistance | **-0.90 (0.38)** | **[-1.66, -0.15]** | 0.43 (0.67) | [-0.88, 1.75] | 1.16 (2.67) | | [-4.10, 6.42] |
| **Model Summary** | *R*^2^ = .21 | | *R*^2^ = .50 | | *R*^2^ = .19 | | |
|  | ***F* (8, 242) = 8.20** | | ***F* (9, 241) = 27.07** | | ***F* (13, 237) = 4.38** | | |
| **Indirect Effects** |  | | **Effect (boot. *SE*)** | | **95%*CI* (bootstrapped)** | | |
| Parental education 🡪 adult SES 🡪 Decrements in eGFR | | |  |  |  |  | |
| Black | | | -1.58 (1.06) |  | [-3.79, 0.41] |  | |
| White | | | -0.56 (0.48) |  | [-1.62, 0.26] |  | |
| Index of moderated mediation | | | -1.02 (1.09) |  | [-3.27, 1.22] |  | |
| Parental education 🡪 EAA GrimAge 🡪 Decrements in eGFR | | |  |  |  |  | |
| Black | | | 0.04 (0.38) |  | [-0.78, 0.85] |  | |
| White | | | -0.47 (0.51) |  | [-1.63, 0.41] |  | |
| Index of moderated mediation | | | 0.52 (0.60) |  | [-0.45, 1.93] |  | |
| Parental education 🡪 adult SES 🡪 EAA GrimAge 🡪 Decrements in eGFR | | | |  |  |  | |
| Black | | | 0.03 (0.20) |  | [-0.40, 0.44] |  | |
| White | | | **-0.32 (0.20)** |  | **[-0.79, -0.05]** |  | |
| Index of moderated mediation | | | **0.34 (0.25)** |  | **[0.01, 0.95]** |  | |

*Note*: *B* = unstandardized regression coefficient, *SE* = standard error; *boot. SE* = bootstrapped standard error, *CI* = confidence interval, EAA = epigenetic age acceleration. Bolded numbers indicate significant association (*p* < .05).

**Table S11.** Full results from moderated mediation analysis on the mediating role of adult SES and DunedinPACE on the association between parental education and decrements in eGFR across a decade, and whether race moderated these mediations (*N* = 251)

|  | Adult SES | | DunedinPACE | | Changes in eGFR | | |
| --- | --- | --- | --- | --- | --- | --- | --- |
|  | *B* (*SE*) | 95%*CI* | *B* (*SE*) | 95%*CI* | *B* (*SE*) | | 95%*CI* |
| Constant | 0.74 (1.50) | [-2.20, 3.69] | -0.09 (0.07) | [-0.23, 0.06] | -20.05 (10.28) | | [-40.31, 0.21] |
| Parental education | **0.83 (0.30)** | **[0.23, 1.42]** | -0.03 (0.02) | [-0.06, 0.00] | -3.03 (2.08) | | [-7.13, 1.07] |
| Adult SES |  |  | **-0.01 (0.00)** | **[-0.02, -0.00]** | -0.74 (0.54) | | [-1.80, 0.33] |
| EAA GrimAge |  |  |  |  | **34.02 (10.81)** | | **[12.73, 55.32]** |
| Race |  |  |  |  | -0.26 (3.10) | | [-6.37, 5.85] |
| Adult SES*Race |  |  |  |  | -0.88 (1.07) | | [-2.99, 1.23] |
| DunedinPACE*Race |  |  |  |  | -29.02 (17.23) | | [-62.96, 4.92] |
| Age | 0.01 (0.02) | [-0.02, 0.04] | -0.00 (0.00) | [-0.00, 0.00] | 0.08 (0.11) | | [-0.13, 0.29] |
| Sex | **-0.83 (0.28)** | **[-1.38, -0.27]** | -0.02 (0.01) | [-0.05, 0.01] | 3.46 (1.96) | | [-0.40, 7.32] |
| Follow-up | -0.04 (0.11) | [-0.26, 0.18] | 0.01 (0.01) | [-0.01, 0.02] | **2.53 (0.75)** | | **[1.05, 4.01]** |
| Smoking | **-1.58 (0.36)** | **[-2.29, -0.87]** | **0.11 (0.02)** | **[0.08, 0.15]** | -5.08 (2.73) | | [-10.47, 0.30] |
| Obese | **-0.99 (0.30)** | **[-1.56, -0.42]** | **0.06 (0.01)** | **[0.03, 0.09]** | 2.87 (2.09) | | [-1.25, 6.98] |
| Elevated blood pressure | 0.04 (0.29) | [-0.54, 0.61] | **0.04 (0.01)** | **[0.02, 0.07]** | -2.82 (2.05) | | [-6.86, 1.21] |
| Insulin resistance | **-0.86 (0.38)** | **[-1.61, -0.12]** | **0.04 (0.02)** | **[0.00, 0.08]** | 1.44 (2.66) | | [-3.80, 6.68] |
| **Model Summary** | *R*^2^ = .21 | | *R*^2^ = .37 | | *R*^2^ = .19 | | |
|  | ***F* (8, 242) = 8.20** | | ***F* (9, 242) = 15.69** | | ***F* (13, 238) = 4.16** | | |
| **Indirect Effects** |  | | **Effect (boot. *SE*)** | | **95%*CI* (bootstrapped)** | | |
| Parental education 🡪 adult SES 🡪 Decrements in eGFR | | |  |  |  |  | |
| Black | | | -1.34 (1.06) |  | [-3.50, 0.73] |  | |
| White | | | -0.61 (0.49) |  | [-1.74, 0.18] |  | |
| Index of moderated mediation | | | -0.73 (1.09) |  | [-2.86, 1.61] |  | |
| Parental education 🡪 EAA GrimAge 🡪 Decrements in eGFR | | |  |  |  |  | |
| Black | | | -0.14 (0.55) |  | [-1.40, 0.88] |  | |
| White | | | -0.92 (0.59) |  | [-2.24, 0.06] |  | |
| Index of moderated mediation | | | 0.79 (0.68) |  | [-0.22, 2.39] |  | |
| Parental education 🡪 adult SES 🡪 EAA GrimAge 🡪 Decrements in eGFR | | | |  |  |  | |
| Black | | | -0.04 (0.17) |  | [-0.46, 0.24] |  | |
| White | | | -0.28 (0.16) |  | [-0.66, -0.05] |  | |
| Index of moderated mediation | | | 0.24 (0.18) |  | [-0.05, 0.66] |  | |

*Note*: *B* = unstandardized regression coefficient, *SE* = standard error; *boot. SE* = bootstrapped standard error, *CI* = confidence interval, EAA = epigenetic age acceleration. Bolded numbers indicate significant association (*p* < .05).

Supplemental Figures


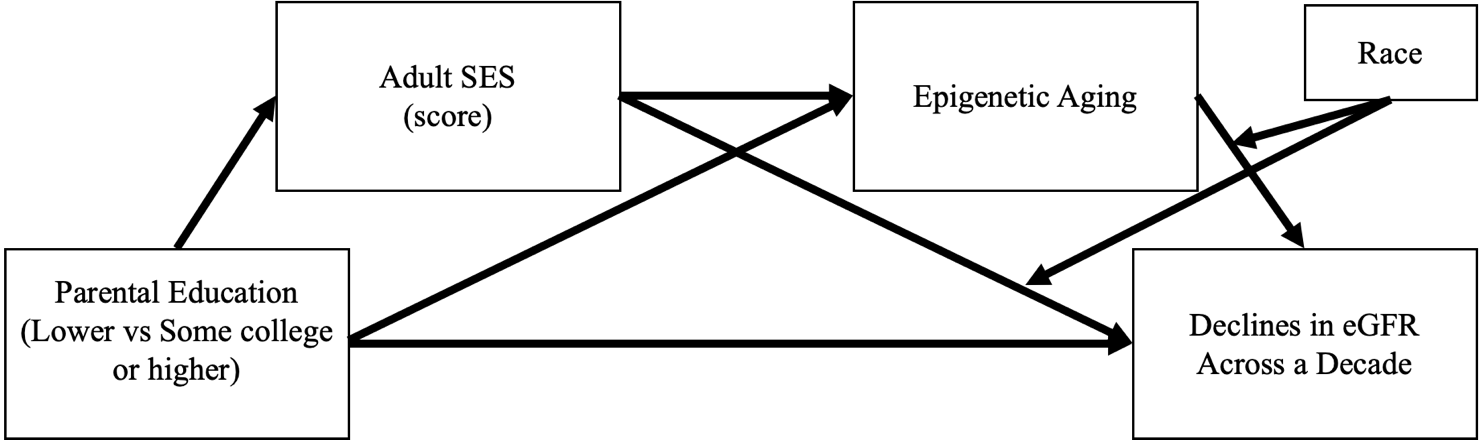


Decrements in eGFR Across a Decade

**Figure S1**. Hypothesized moderated mediation model of the mediating role of adult SES and epigenetic aging on the association between parental education and decrements in eGFR across a decade and whether race moderated these mediations.


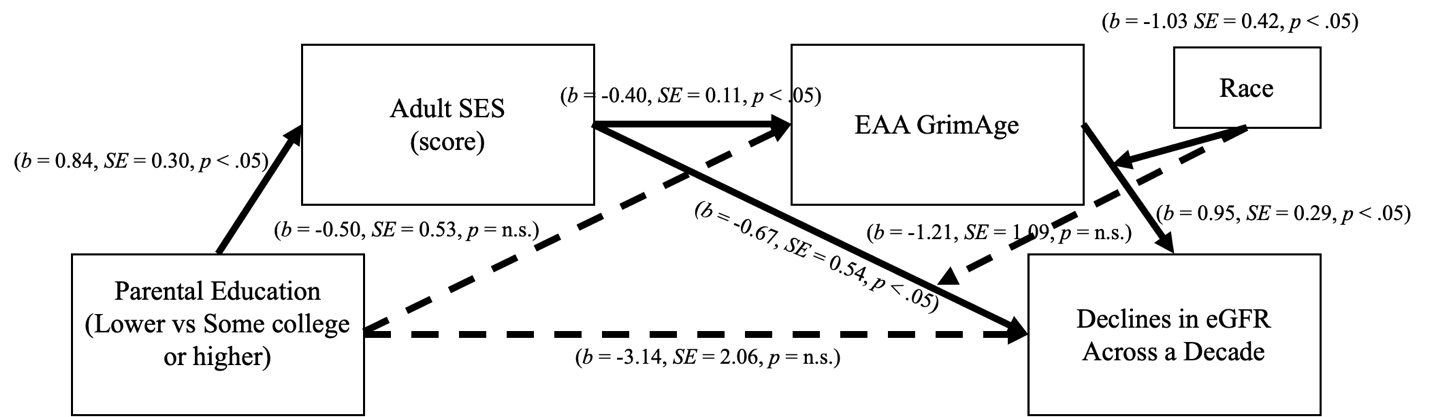


Decrements in eGFR Across a Decade

**Figure S2**. Results from the moderated mediation model of the mediating role of adult SES and EAA GrimAge on the association between parental education and decrements in eGFR across a decade and whether race moderated these mediations. Race moderated the association between EAA GrimAge and changes in eGFR across a decade.
